# Heterologous production of the widely used natural food colorant carminic acid in *Aspergillus nidulans*

**DOI:** 10.1038/s41598-018-30816-9

**Published:** 2018-08-27

**Authors:** Rasmus J. N. Frandsen, Paiman Khorsand-Jamal, Kenneth T. Kongstad, Majse Nafisi, Rubini M. Kannangara, Dan Staerk, Finn T. Okkels, Kim Binderup, Bjørn Madsen, Birger Lindberg Møller, Ulf Thrane, Uffe H. Mortensen

**Affiliations:** 1 Section for Synthetic Biology, Department of Biotechnology and Biomedicine, The Technical University of Denmark, Kongens Lyngby, Denmark; 2 Chr. Hansen Natural Colors A/S, Hoersholm, Denmark; 3 Novo Nordisk A/S, Maaloev, Denmark; 4 Department of Drug Design and Pharmacology, University of Copenhagen, Copenhagen, Denmark; 5 Plant Biochemistry Laboratory, Department of Plant and Environmental Sciences, University of Copenhagen, Frederiksberg, Denmark; 6 Center for Synthetic Biology, University of Copenhagen, Frederiksberg, Denmark; 7 Present Address: Actabio ApS, Roskilde, Denmark; 8 Present Address: DSM Nutritional Products, Kaiseraugst, Switzerland; 9 Present Address: Department of Energy Performance, Indoor Environment and Sustainability, Danish Building Research Institute, Aalborg University Copenhagen, Copenhagen, Denmark; 10 Present Address: River Stone Biotech ApS, København Ø, Fruebjergvej 3, 2100 Denmark

## Abstract

The natural red food colorants carmine (E120) and carminic acid are currently produced from scale insects. The access to raw material is limited and current production is sensitive to fluctuation in weather conditions. A cheaper and more stable supply is therefore desirable. Here we present the first proof-of-concept of heterologous microbial production of carminic acid in *Aspergillus nidulans* by developing a semi-natural biosynthetic pathway. Formation of the tricyclic core of carminic acid is achieved via a two-step process wherein a plant type III polyketide synthase (PKS) forms a non-reduced linear octaketide, which subsequently is folded into the desired flavokermesic acid anthrone (FKA) structure by a cyclase and a aromatase from a bacterial type II PKS system. The formed FKA is oxidized to flavokermesic acid and kermesic acid, catalyzed by endogenous *A*. *nidulans* monooxygenases, and further converted to dcII and carminic acid by the *Dactylopius coccus* C-glucosyltransferase DcUGT2. The establishment of a functional biosynthetic carminic acid pathway in *A*. *nidulans* serves as an important step towards industrial-scale production of carminic acid via liquid-state fermentation using a microbial cell factory.

## Introduction

Carminic acid is a widely used natural red food colorant, which belongs to the coccid dye family which also includes flavokermesic acid, kermesic acid and laccaic acids^1^. These compounds all share the same anthraquinone core and are produced by various scale insect species, including *Dactylopius coccus* (Mexican cochineal)^2^. Coccid dyes have been utilized for textile dying, cosmetics and food for at least 2800 years, and the production methods have not changed significantly throughout history^2,3^. Current industrial production is estimated to be 800 tons per year made by facilities that are mainly located in Peru, Canary Islands, Chile and Mexico. Here, *D*. *coccus* is collected from populations of insects living on *Opuntia* cacti, which are either grown in large plantations or in their natural habitats^4^. Moreover, extraction and purification of carminic acid from *D*. *coccus* depends a complex multistep process^5^. Production is therefore expensive, cumbersome, and vulnerable to weather fluctuations and to attacks by plant and insect pathogens^6^. A cheap, simple and stable production is therefore desirable and could potentially be achieved by heterologous microbial production.

**Figure 1:**
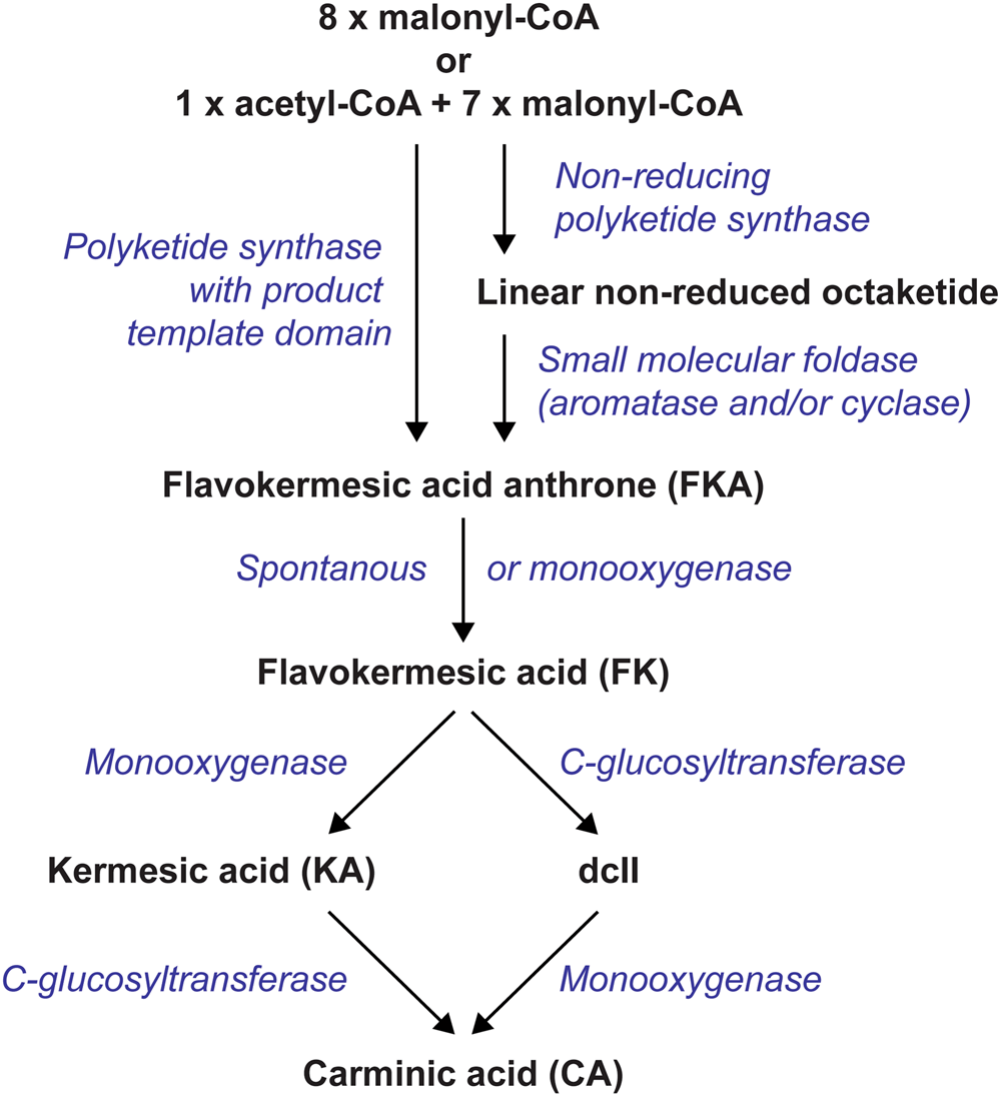
Carminic acid biosynthesis. Proposed biosynthetic pathway for formation of carminic acid and the required enzymatic steps. Formation of flavokermesic acid anthrone can theoretically be achieved via either a one-step (left) or two-step (right) process.

Towards construction of a microbial cell factory for carminic acid production, we have previously shown that the first intermediate in the carminic acid synthesis is the polyketide flavokermesic acid^7^ and that the glucose moiety of carminic acid is added by an endoplasmatic reticulum (ER) membrane-bound C-glucosyltransferase, UGT2^8^. Based on that, we have presented a five-step biosynthesis model for the natural process in *D*. *coccus*^9^ (Fig. 1), which depends on one polyketide synthase (PKS), one or two monooxygenases and a C-glucosyltransferase. However, the enzymatic basis for formation of the first intermediate in the natural pathway, flavokermesic acid anthrone, and the subsequent monooxygenation steps remain unknown. The involvement of a PKS in the pathway is particularly enigmatic since no functional animal PKSs have ever been identified. Hence, the PKS may be encoded by a gene in an endosymbiont rather than by a gene in the genome of the insect^4,10^. However, neither the recently released partial *D*. *coccus* genome sequence^6^ nor the genomes for the *Candidatus Wolbachia bourtzisii* wDacA and *Candidatus Wolbachia pipientis* wDacB endosymbions^5^ unveil any obvious non-reducing PKS or modified fatty acid synthase (FAS) candidates. In the absence of the natural PKS, we have adopted an alternative strategy for constructing a microbial cell factory for carminic acid production. Here we explored the possibility of establishing a semi-natural carminic acid pathway in the filamentous fungus *Aspergillus nidulans*, a well-characterized producer of a wide range of aromatic polyketides, using a synthetic biology approach. Using this strategy we have established proof-of-concept of stable microbial production of carminic acid.

## Results

### Fungal host

Filamentous fungi may serve as hosts for a microbial carminic acid cell factory as they have the capacity to deliver an ample supply of acetyl-CoA and malonyl-CoA building blocks for the synthesis of the polyketide scaffold. They also offer the necessary cellular infrastructure to accommodate the ER membrane bound C-glucosyltransferase UGT2^8^. Since *A*. *nidulans* possesses a well-developed genetic engineering toolbox, we chose this species as a starting point towards the development of a fungal carminic acid cell factory. Like most filamentous fungi, *A*. *nidulans* is able to produce a plethora of biosynthetically diverse secondary metabolites including a significant number of aromatic polyketides. Chiang *et al*. have previously demonstrated that deletion of the gene clusters that are responsible for formation of the major endogenous PKS products in *A*. *nidulans*^11^, improved the potential of *A*. *nidulans* as a cell factory for heterologous production of polyketides. In this way, the pools of acetyl-CoA and malonyl-CoA building blocks are increased, and the subsequent chemical analyses for the formation of new products simplified. As a first step towards a fungal cell factory for carminic acid production, we therefore eliminated the biosynthetic pathways for the dominating aromatic polyketides that could potentially interfere with carminic acid production and complicate the analysis and downstream purification. Specifically, the gene clusters for production of asperthecin, monodictyphenone, and sterigmatocystin were eliminated as well as the genes responsible for green conidia pigment formation, *wA* and *yA*, were eliminated in a non-homologous endjoining defficient *A*. *nidulans* background. Besides the expected lack of conidial pigments, the resulting strain, NID2252, did not display any visible effects on morphology and fitness (Supplementary File, Fig. 1). We therefore used NID2252 as a reference strain and as the basis for the construction of a fungal cell factory for carminic acid production.

### Design of a semi-natural pathway for flavokermesic acid anthrone formation

The major roadblock for the construction of a microbial carminic acid cell factory was lack of a natural PKS that synthesizes flavokermesic acid anthrone, the first putative intermediate in the pathway. To bypass this limitation, we pursued an alternative biosynthetic route for production of this polyketide. The octaketide flavokermesic acid anthrone, with its C7-C12, C5-C14 and C2-C15 cyclization pattern, could in theory be formed in a single step by a fungal non-reducing type I iterative PKS that possesses a suitable product template domain (Fig. 1 left). However, no such enzymes have yet been described in the literature. An alternative mechanism for forming the flavokermesic acid anthrone is via a two-step reaction where a PKS synthesizes a non-reduced linear octaketide chain, which subsequently is cyclized into the desired structure by trans-acting cyclases/aromatases (Fig. 1 right). Reactions of this type exist in nature in e.g. the actinorhodin (Act) pathway in *Streptomyces coelicolor*^12^. In this case, the octaketide backbone is synthesized by a multi-subunit bacterial type II PKS system, KS_α_, KS_β_ and ACP (the *act* minimal PKS), which is reduced by *act*KR at position C9 and subsequently folded by the aromatase/cyclase, *act*ARO/CYC and cyclase *act*CYC2, to yield the tricyclic compound 3,8-dihydroxy-1-methylanthraquinone-2-carboxylic acid (DMAC)^7^. DMAC displays the same fold as flavokermesic acid anthrone but is reduced at the C9 position.

**Figure 2:**
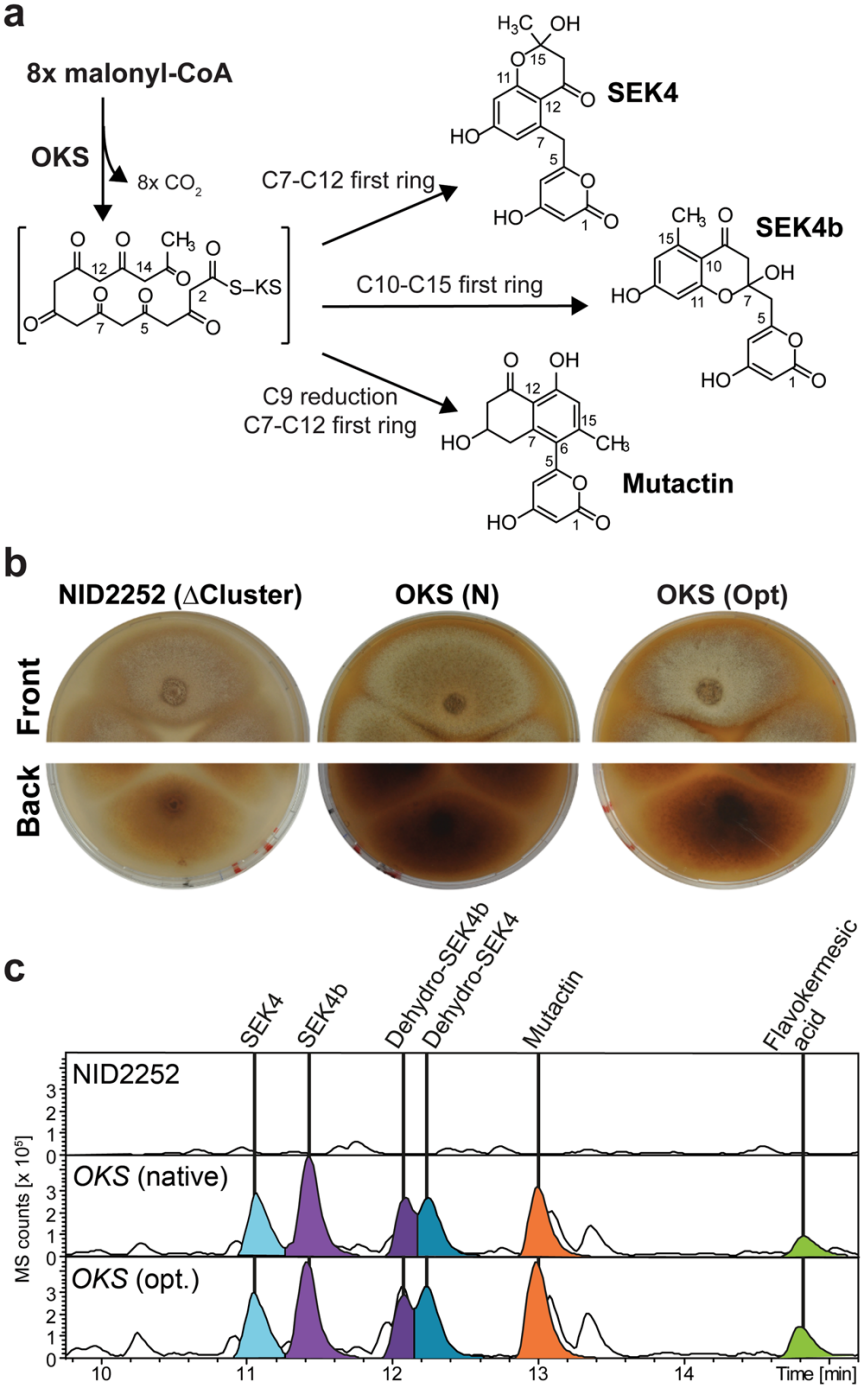
Formation of the non-reduced octaketide. (**a**) Spontaneous folding of non-reduced octaketides. (**b**) Phenotype of *Aspergillus nidulans* NID2252 reference strain and strains that express *OKS* either with native or optimized codon usage. (**c**) Metabolic profile of *A*. *nidulans* NID2252 reference strain and *OKS* expressing strains. Base peak chromatogram (light grey) with indication of selected metabolites: SEK4 (blue), dehydro-SEK4 (dark blue), SEK4b (purple), dehydro-SEK4b (dark purple), mutactin (orange), and flavokermesic acid (lime).

Of special interest for the present study, is the ZhuI cyclase and ZhuJ aromatase from *Streptomyces* sp. R1128, which catalyze two sequential cyclization reactions of non-reduced linear polyketides into folds similar to that of flavokermesic acid an throne^13^. Specifically, the ZhuI cyclase is responsible for closing the first ring, C7-C12, and the ZhuJ aromatase for closing the second ring, C5-C14. The third ring, C2-C15, is formed spontaneously. *ZhuI* and *ZhuJ* have previously been co-expressed in *S*. *coelicolor* with the octaketide forming type II *act* minimal PKS resulting in the formation of flavokermesic acid, known as 3,6,8-trihydroxy-1-methylanthraquinone-2-carboxylic acid (TMAC) in the bacterial literature^14^. Unfortunately, an identical strategy may be difficult to implement in *A*. *nidulans* as the type II minimal PKSs have not been successfully implemented in heterologous hosts outside the *Streptomyces* genus despite several attempts^15^. And indeed our attempts to reconstitute the *act* miniPKS in *A*. *nidulans* and *Saccharomyces* *cerevisiae* similarly proved unsuccessful (data not shown). An alternative way for forming the required non-reduced octaketide is to rely on type III PKS enzymes, which have been successfully expressed in yeast, e.g. in connection with flavonoid biosynthesis^16^. Of special interest is the *Aloe arborescens* octaketide synthase (OKS), which has been described to produce non-reduced octaketides that spontaneously folds into SEK4 and SEK4b *in vitro*^17^ (Fig. 2a). However, in  *Aspergillus* it remained untested whether the products of type III PKSs, which are ACP-independent enzymes that release carboxylic acids, can be folded by the ZhuI type of cyclase that has been described to act on ACP bound substrates. In the current study we hence set out to test the compatibility of the plant type III OKS with that of cyclases/aromatases from bacterial type II PKS systems, with the aim of developing an artificial *de novo* biosynthetic pathway for formation of the flavokermesic acid anthrone precursor for formation of carminic acid.

### Production of polyketide precursors for carminic acid production in *A*. *nidulans*

To test the *in vivo* performance of OKS in a fungal host, we inserted *OKS* controlled by the *A*. *nidulans gpdA* promoter as a single copy into a defined locus (integration site 5, IS5) in the *A*. *nidulans* genome^18^. Two strains were constructed, one expressing the native *OKS* coding sequence and one expressing a codon optimized version for expression in *A*. *nidulans*. Following seven days of incubation on solid growth media, see the Methods section for details, both strains displayed a strong red-brown coloring of the mycelium (Fig. 2b). Metabolic profiling by HPLC-HRMS of the strains did not reveal any traces of a non-reduced octaketide, but rather the expected SEK4 and SEK4b along with flavokermesic acid and three other abundant compounds of unknown identity (Fig. 2c), which were not present in the NID2252 reference strain. SEK4 and SEK4b were identified based on thorough HPLC-HRMS/MS analysis (Supplementary File, Fig. 2) and was in accordance with previously reported values^19^ while flavokermesic acid was compared to an authentic reference isolated from *D*. *coccus*^9^ (Supplementary File, Fig. 3). The metabolite eluting at 13.0 min. had a pseudomolecular ion [M + H]^+^ of *m*/*z* 303.0867, corresponding to a molecular formula of C_16_H_14_O_6_, consistent with the known octaketide shunt product, mutactin, previously described in *Streptomyces* based experiments with the *Act* gene cluster^7^. This identification was confirmed through detailed analysis of the UV spectrum, and supported by the retention time relative to SEK4 and SEK4b (Supplementary File, Fig. 4). The two additional metabolites with molecular formulas of C_16_H_12_O_6_ ([M-H]^−^ *m*/*z* 299.0566) indicated octaketide products but did, however, not correspond to a common shunt product (Supplementary File, Fig. 5). Hence, the metabolites were isolated and structure elucidation based on 1D and 2D NMR experiments identified them as dehydro-SEK4 (also known as B26 in the bacterial literature) and dehydro-SEK4b^20^ (Supplementary File, Figs 6–11). Formation of the six new polyketides observed in the *OKS* strain can be explained by three different spontaneous first ring closure reactions: C7-C12 (SEK4, dehydro-SEK4 and flavokermesic acid), C10-C15 (SEK4b and dehydro-SEK4b) and C7-C12 after C9 ketone reduction (mutactin) (Fig. 2a and Supplementary File, Fig. 12). Of these, only the first ring closure type (C7-C12) allows for the subsequent formation of the desired flavokermesic acid anthrone via a second C5-C14 and third C2-C15 ring closure. SEK4 and SEK4b have previously been reported to spontaneously dehydrate to form dehydro-SEK4 and dehydro-SEK4b, respectively, while formation of mutactin depends on enzymatic reduction of the C9 position of the polyketide chain prior to folding^21^. The formation of flavokermesic acid, a known intermediate in the carminic acid pathway (Fig. 1), was unexpected as this metabolite has not been reported to form spontaneously from non-reduced octaketide chains. This suggests that *A*. *nidulans* naturally produces a number of cyclases/aromatases that promote the desired folding pattern. The metabolite profiling of the two *OKS* expressing strains did not show any substantial differences in production levels and we decided to focus on the strain expressing the native version of *OKS* as basis for further developments of a fungal cell factory for carminic acid production.

### Engineering the folding pathway of non-reduced octaketides

**Figure 3:**
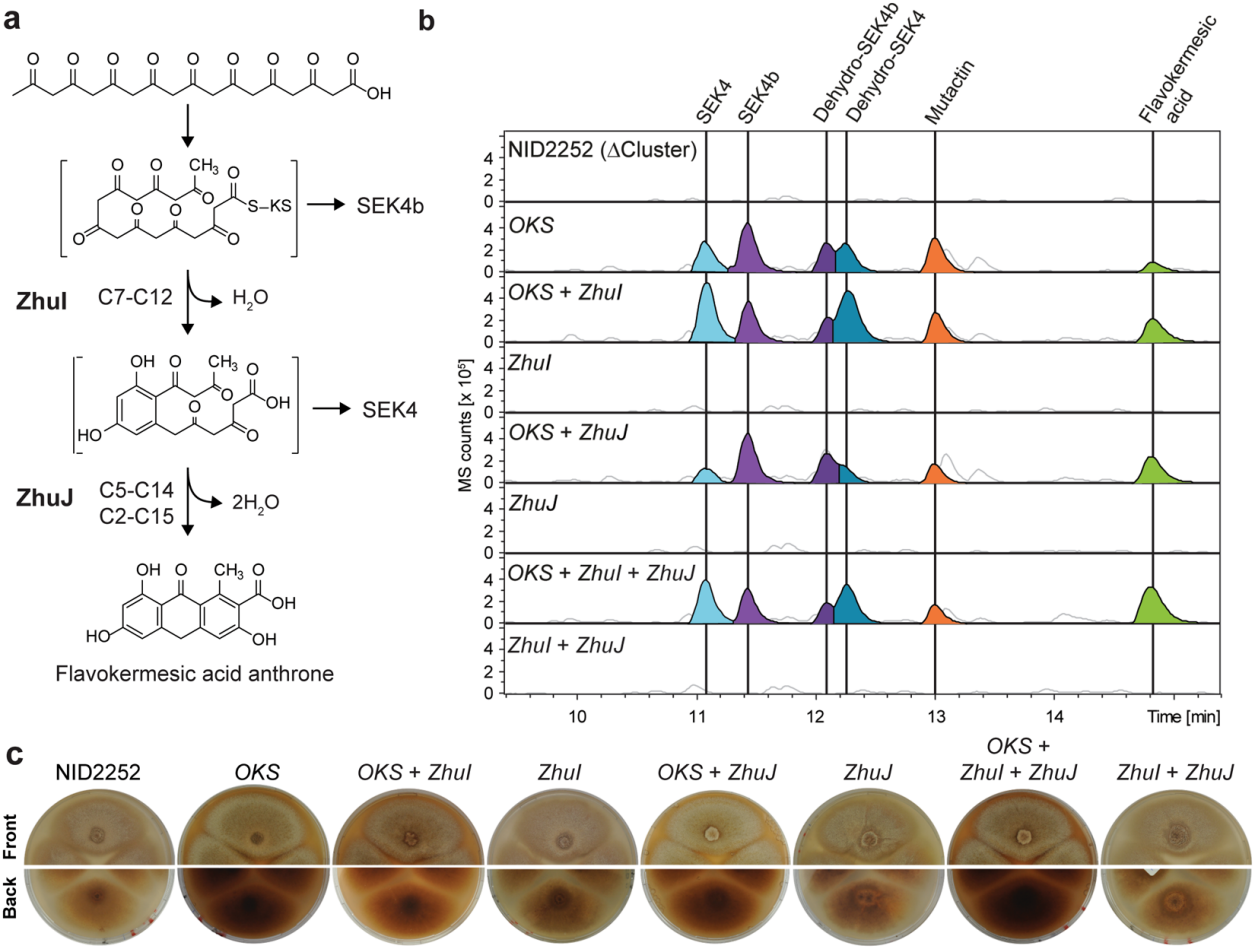
Directing folding of the octaketide backbone. (**a**) Biosynthetic steps from non-reduced octaketide to flavokermesic acid anthrone. (**b**) Targeted chemical analysis of the *Aspergillus nidulans* NID2252 reference strains (strain with deleted PKS clusters), and NID2252 expressing *ZhuI*, or *ZhuJ*, or *ZhuI* + *ZhuJ*, or *OKS*, or *OKS* + *ZhuI*, or *OKS* + *ZhuJ* and *OKS* + *ZhuI* + *ZhuJ*. Base peak chromatogram (light grey) with indication of selected metabolites: SEK4 (blue), dehydro-SEK4 (dark blue), SEK4b (purple), dehydro-SEK4b (dark purple), mutactin, (orange) and flavokermesic acid (lime). (**c**) Colony morphology of strains propagated for seven days on minimal medium.

The promiscuous folding of the non-reduced octaketide reduces the yield of desirable flavokermesic acid. We therefore envisioned that flavokermesic acid production could be increased by streamlining the folding process in the desired direction by expressing codon optimized versions of *ZhuI* and *ZhuJ*. To examine whether ZhuI and ZhuJ interfere with the native metabolism of *A*. *nidulans*, we first constructed strains expressing *ZhuI* and *ZhuJ* individually and in combination from defined genomic loci (IS6 and IS7) in the *A*. *nidulans* NID2252 reference strain. Metabolic profiling by HPLC-HRMS of the three strains did not uncover any detectable metabolic differences (Fig. 3b). We then constructed a strain co-expressing *OKS* with *ZhuI*, which encodes the cyclases that catalyzes formation of the C7-C12 first ring closure (Fig. 3a). As compared to the strain expressing only *OKS*, this strain showed 2.5-, 2.2-, and 2.1-fold increases in flavokermesic acid, SEK4, and dehydro-SEK4 levels, respectively. In agreement with *ZhuI* guiding folding in the desired direction, these metabolic changes were accompanied by reduced levels of metabolites containing the undesired first ring folds (Fig. 3b and Table 1). A strain co-expressing *OKS* and *ZhuJ*, which encodes the aromatases that catalyzes the C5-C14 second ring closure (Fig. 3a), produced a 2.7-fold increase in flavokermesic acid levels. This increase was accompanied by a reduction in the levels of metabolites with a C7-C12 first ring fold (SEK4 and dehydro-SEK4), while, as expected the level of metabolites with the undesired C10-C15 first ring fold (SEK4b and dehydro-SEK4b) were not affected (Fig. 3b and Table 1). Finally, analysis of the strain expressing *ZhuI*, *ZhuJ*, and *OKS* displayed a 4.1-fold increase in the production of flavokermesic acid compared to when *OKS* was expressed alone. Moreover, this increase is 60% higher than what was observed with the strains expressing *OKS* in combination with either *ZhuI or ZhuJ*, respectively (Table 1). This result indicates that the cyclase and aromatase, in an additive manner, are able to increase the flux in the artificial *de novo* pathway towards the desired product, flavokermesic acid (Fig. 3b).

**Table 1:** Normalized levels of metabolites in *OKS*, *ZhuI* and *ZhuJ* expressing strains.

	*OKS*	*OKS* + *ZhuI*	*OKS* + *ZhuJ*	*OKS* + *ZhuI* + *ZhuJ*
SEK4	3.8 ± 0.2 (1)	8.2 ± 1.0 (2.2)	0.9 ± 0.2 (0.2)	6.0 ± 0.3 (1.6)
SEK4b	6.8 ± 0.7 (1)	5.2 ± 0.6 (0.8)	6.4 ± 0.7 (0.9)	4.6 ± 0.3 (0.7)
dehydro-SEK4	4.6 ± 0.4 (1)	9.5 ± 0.9 (2.1)	2.28 ± 0.09 (0.5)	6.9 ± 0.7 (1.5)
dehydro-SEK4b	3.6 ± 0.3 (1)	2.5 ± 0.2 (0.7)	4.1 ± 0.3 (1.1)	2.4 ± 0.2 (0.7)
Mutactin	4.8 ± 0.2 (1)	3.6 ± 0.6 (0.8)	2.2 ± 0.4 (0.5)	2.5 ± 0.2 (0.5)
Flavokermesic acid	1.8 ± 0.3 (1)	4.5 ± 0.4 (2.5)	4.9 ± 0.6 (2.7)	7.6 ± 0.6 (4.1)
SEK4:SEK4b	0.56 ± 0.02	1.59 ± 0.01	0.14 ± 0.02	1.31 ± 0.01
dehydro-SEK4:dehydro-SEK4b	1.26 ± 0.06	3.72 ± 0.15	0.56 ± 0.02	2.9 ± 0.2

The base peak ion count normalized to linoleic acid (biomass) levels in the individual samples (*n* = 3). Values in parentheses indicate the metabolite level relative to the level of the same metabolite obtained with the strain expressing *OKS* only.

**Figure 4:**
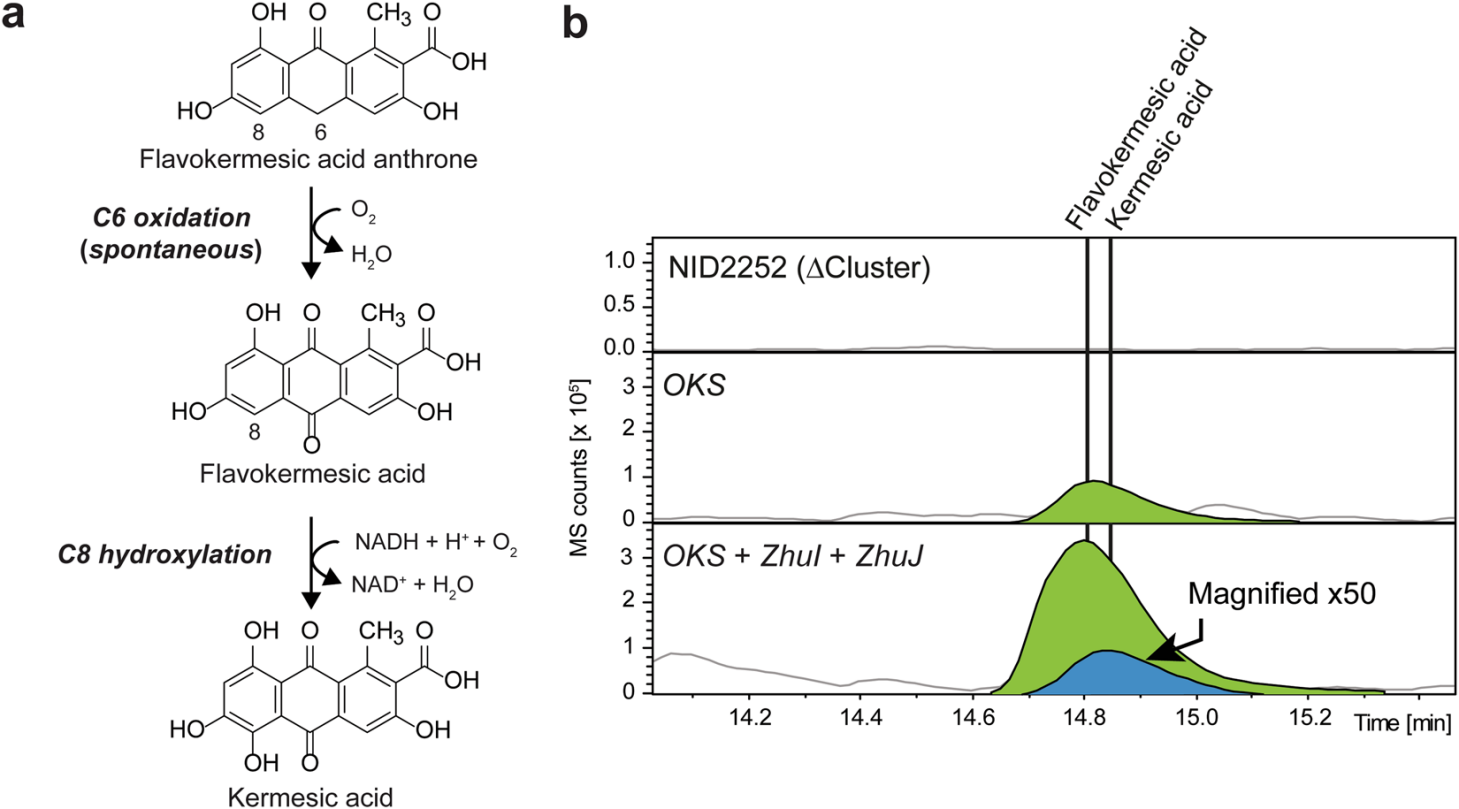
Oxidative steps involved in the formation of kermesic acid. (**a**) Oxidative steps from flavokermesic acid anthrone to kermesic acid. (**b**) Targeted metabolic analysis for the production of flavokermesic acid (FK) and kermesic acid (KA) in the NID2252 reference strain (strain with deleted PKS clusters), *OKS* expressing strain and the *OKS* + *ZhuI* + *ZhuJ* strain. The chromatogram shows the base peak chromatogram (light grey) with indication of flavokermesic acid (lime) and kermesic acid (blue).

Surprisingly, and very encouraging, further metabolic analysis of the strain co-expressing *OKS, ZhuI* and *ZhuJ* revealed that the increased formation of flavokermesic acid was accompanied by the production of kermesic acid (Fig. 4). Hence, *A*. *nidulans* provides the necessary monooxygenase for converting flavokermesic acid into kermesic acid, which may serve as the final intermediate in the carminic acid pathway (Fig. 1).

### Production of carminic acid in *A*. *nidulans*

**Figure 5:**
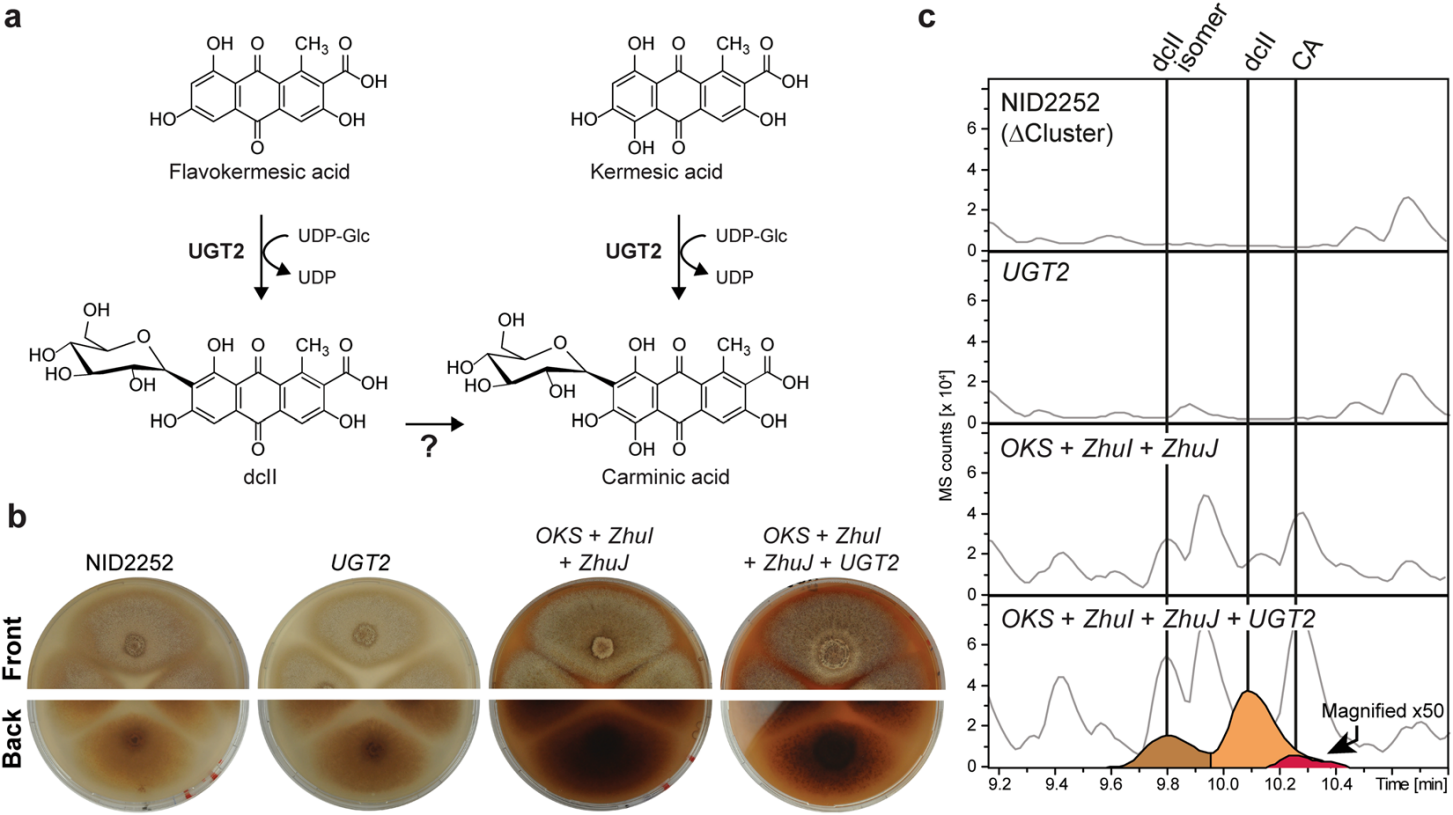
Glucosylation step for forming carminic acid. (**a**) The *Dactylopius coccus* glucosyltransferase UGT2 accepts both kermesic acid and flavokermesic acid as substrates, resulting in the formation of carminic acid and dcII, respectively. (**b**) The phenotypical effect of expressing *UGT2* alone and in combination with the synthetic PKS in the NID2252 *Aspergillus nidulans* background, pictures taken after seven days of incubation. (**c**) Targeted metabolic analysis for the production of glucosylated compounds, e.g. carminic acid (red) and dcII (orange) overlaid base peak chromatogram (light grey).

The last step in carminic acid formation involves C-glucosylation of kermesic acid (Fig. 5a). The responsible enzyme for this step in the carminic acid biosynthetic pathway has previously been identified in *D*. *coccus* and characterized by heterologous expression in *S*. *cerevisiae*^8^. We therefore inserted the UGT2 encoding gene, optimized for expression in *A*. *nidulans*, into the IS4 locus of the NID2252 reference strain, and to complete the semi-natural carminic acid biosynthetic pathway in *A*. *nidulans*, into the same locus in the strain co-expressing *ZhuI*, *ZhuJ*, and *OKS*. Expression of *UGT2* alone in the reference strain did not affect the appearance of the growth medium color nor the metabolic profile of *A*. *nidulans* when compared to the NID2252 reference strain (Fig. 5b,c). In contrast, co-expression of *OKS, ZhuI, ZhuJ*, and *UGT2* gave rise to a red coloring of the medium, suggesting that a more water-soluble colorant(s) was formed (Fig. 5b). Targeted LC-HRMS analysis of this strain confirmed the formation of carminic acid^22^ (Supplementary File, Fig. 13), along with dcII^9,23^ and a dcII isomer (Supplementary File, Fig. 14), showing that UGT2 was functional in *A*. *nidulans* and could successfully complete the biosynthetic pathway (Fig. 5c). Positive identification of carminic acid and dcII was based on similar retention times, UV-spectra, MS and MS/MS fragmentation patterns for pure standards of the two compounds. Three isomers of carminic acid have been described in literature (carminic acid, DCIV and DCVII) by Stathopoulou *et al*.^23^, all three show different retention times in LC-MS/MS based analysis. In our analysis we only detected carminic acid and not DCIV and DCVII based on retention time and fragmentation pattern of authentic carminic acid standard isolated from *D*. *coccus* (Supplementary file, Fig. 13). Together our data firmly demonstrate that it is possible to produce carminic acid in *A*. *nidulans* based on a semi-natural pathway.

## Discussion

The results presented here represent the first proof of principle for the functionality of a novel artificial biosynthetic pathway that allows for microbial production of carminic acid. A major challenge towards this feat was the lack of a natural PKS that delivers the polyketide scaffold of carminic acid. In our *A*. *nidulans* cell factory, we solved this problem by co-expressing genes encoding a type III plant PKS, bacterial cyclase and a bacterial aromatase to form an artificial three component PKS system that was demonstrated to be functional *in vivo*. The fact that type III PKSs are acyl carrier protein (ACP) independent enzymes that release their products as free carboxylic acids^24^ makes this finding quite surprising considering that both *in vitro* and crystallographic analysis of cyclases and aromatases from type II PKS systems suggest that they only act on ACP-bound intermediates^25^. A possible explanation for the observed functionality could be that the cyclase and aromatase are produced at very high levels in *A*. *nidulans*, which might compensate for suboptimal biochemistry. Alternatively the continued accumulation of the five shunt products (Fig. 3b) may be explained by low cyclase and aromatase levels in the cell, which result in low folding kinetics, allowing part of pool of non-reduced linear polyketides to fold spontaneously. s  Previously, heterologous transient expression of the synthetic PKS system in *Nicotiana benthamiana* (tobacco) demonstrated that the PKS system produce similar compounds under these conditions^26^. Thus this artificial biosynthetic pathway may be generally applicable for synthesis of carminic acid in a multiplicity of heterologous hosts.

The developed artificial type III/type III PKS system offers a generally applicable synthetic biological solution for the formation of both natural and novel compounds with aromatic motifs. An area of special interest are the natural occurring bacterial type II PKS biosynthetic pathways found in *Streptomyces* sp., which in general have proven nearly impossible to be functionally expressed in standard production host organisms such as *S*. *cerevisiae* and *E*. *coli*. The presented solution for the first time offers a workaround for these troublesome PKS systems. We envision, for example, the production of the antibiotic actinorhodin in a non-*Streptomyces* host, by co-expressing *OKS* with genes encoding the folding and tailoring activities from the natural *act* pathway, circumventing the troublesome *act* minimal PKS step. The new PKSII/III system may also prove a new and faster avenue for characterization of the many novel type II PKS operons identified by genome sequencing projects. Moreover, it has not escaped our notice that the artificial PKS system also forms the basis for implementation of the first ever programmable PKS platform for the formation of aromatic compounds, offering control over the carbon chain length by choice of the used PKS, and the carbon backbone folding pattern via the deployed cyclase(s) and aromatase(s). Such a platform would allow for the formation of both novel molecular scaffolds, not found in nature, as well as the construction of novel *de novo* biosynthetic pathways for known natural aromatic compounds, in cases where the natural biosynthetic pathway is currently unknown.

Surprisingly, co-expression of *OKS*, *ZhuI* and *ZhuJ* in *A*. *nidulans* resulted in the formation of flavokermesic acid, and kermesic acid, and not the expected flavokermesic acid anthrone. Monooxygenation of the central C6 position in the flavokermesic acid anthrone (Fig. 4a) has previously been observed in *S*. *coelicolor* strains co-expressing the *act* minimal PKS with *ZhuI* and *ZhuJ*. Tang *et al*.^14^ have speculated that the reaction proceeds spontaneously driven by the formation of the energetically more favourable anthraquinone structure. We have previously observed rapid spontaneous oxidation of chemically synthesized flavokermesic acid anthrone^9^ supporting this view. In contrast, we note that flavokermesic acid generated in *S*. *coelicolor* by the *act* minimal PKS, ZhuI and ZhuJ does not appear to oxidize to kermesic acid spontaneously. This finding suggests that *A*. *nidulans* contains an endogenous enzyme(s) that can catalyze the C8 hydroxylation of flavokermesic acid to yield kermesic acid. The responsible enzyme likely catalyzes a similar reaction in one of the endogenous biosynthetic pathways of *A*. *nidulans* that leads to the formation of anthraquinone metabolites. Similar hydroxylation reactions have been documented in several fungal PKS biosynthetic pathways^27^ and typically rely on flavin-dependent monooxygenases (FMO) or P450 cytochrome monooxygenases.

Completion of the carminic acid biosynthetic pathway in *A*. *nidulans* was achieved by introducing the previously identified *D*. *coccus* UGT2 encoding gene into the flavokermesic acid and kermesic acid producing *A*. *nidulans* strain, resulting in the formation of dcII and carminic acid. Previous *in vitro* characterization of the *D*. *coccus* UGT2 has shown that this enzyme accepts both flavokermesic acid and kermesic acid as substrates, resulting in the formation of dcII and carminic acid, respectively (Fig. 5a), both metabolites that are also known to accumulate in *D*. *coccus*^23^. As specified in Fig. 1 the order of the last two enzymatically catalyzed steps in carminic acid biosynthesis is unresolved and it is currently unknown whether only one of them, or both, are functional *in vivo*. In addition to the two expected glucosylated polyketides we also observed accumulation of a dcII isomer with a MS/MS fragmentation pattern characteristic for C-glucosylation (Fig. 5b and Supplementary File, Fig. 3). Retention time relative to dcII and abundance of individual MS/MS fragments were, interestingly, similar to that of the proposed dcI metabolite observed in *D*. *coccus* by Lech and co-workers^28^. This is quite surprising as *in vitro* characterization of UGT2 suggests that it is quite substrate specific both with respect to the aglycon and sugar-donor^8^. Lech and co-workers suggest a dcII isomer with a different substitution pattern on the aglycon, namely hydroxylations on either C-3, C-5 and C-6 or C-5, C-6 and C-8, based on relative retention times of the related anthraquinones alizarin, xanthopurpurin, and quinizarin. Other researchers have, in *D*. *coccus*, observed carminic acid to be in equillibrium with C-1′-epimers of the furanose analogue, acompagnied by a markedly increase in retention time^23^. Thus, the similar retention times of dcII and the dcII isomer suggest both to be in the form of pyranose. However, unambiguous identification of the structure of the dcII isomer requires a detailed NMR analysis which was not possible due to insufficient amounts.

Since significant amounts of red pigments were un-extractable from the mycelium, the overall efficiency of the cell factory for production of carminic acid cannot easily be assessed. However, the fraction of soluble carminic acid in the medium is far from the levels necessary for commercial production; and further optimization is therefore essential. Firstly, the endogenous *A*. *nidulans* monooxygenase acting on flavokermesic acid and/or on dcII is limiting. The gene encoding this activity, therefore, needs to be identified and overexpressed to remove this bottleneck in the pathway. However, the *A*. *nidulans* genome includes 54 FMO and 122 P450 encoding genes^29^, meaning that a large effort is necessary to identify the responsible enzyme(s). Successful identification of the responsible gene may also be key towards functional transfer of the pathway to more industrially relevant filamentous fungi like *A*. *niger* or *A*. *oryzae*. Secondly, if elements of the fungal mycelium specifically bind carminic acid, soluble yields of carminic acid can be increased by genetically removing these features if they are not important for fitness. Thirdly, the entire pathway needs to be fine-tuned by identifying the proper ratio of *OKS* expression level relative to that of *ZhuI*, *ZhuJ* and *DcUGT2*. When this ratio is known, more gene copies maintaining this ratio can be inserted to further increase yields. Additional improvements can be achieved via standard metabolic engineering strategies^30^. Hence, our carminic acid cell factory represents the first step towards sustainable and stable production of this classical natural food colorant in bioreactors.

## Methods

### Organisms, media and growth conditions

*Aspergillus nidulans* NID1^31^ was used as the fungal expression host in the current study. The constructed strains are summarized in Supplementary File, Table 1. The fungus was propagated on solid Minimal Medium (MM) supplemented with 10 mM uracil (Ura), 10 mM uridine (Uri) and 4 mM arginine (Arg), at 37 °C in darkness. Following strain construction, the *AfpyrG* marker was removed by counter selection on MM + Ura + Uri + Arg plates supplemented with 7,5 mM 5-Fluoroorotic acid (5-FOA). Plasmids were constructed using chemical competent *E*. *coli* DH5a, grown in Lysogeny Broth (LB) medium supplemented with ampicillin.

### Genetic engineering of *Aspergillusnidulans*

Targeted deletion of the *wA* and *yA* genes were performed using the split-marker PCR-based method, described by Nielsen and co-workers^32^. Targeting sequence, approximately 1500 bp long, for the two loci in the *A*. *nidulans* genome were amplified using the primers described in Supplementary File, Table 2 and X7 DNA polymerase^33^. *A*. *fumigatus pyrG*, surrounded by direct repeats, was used as selection marker and amplified in two overlapping fragments which were fused with the gene targeting fragments by fusion-PCR. The gel-purified pairs of targeting fragments were sequentially transformed into the non-homologous end-joining deficient NID1 background to create NID598 (*wA*∆ and *yA*∆). Following the genetic modification the *AfpyrG* marker was eliminated by counter selection on 5-FOA.

The *Apt*-cluster, *mdp*-cluster and *stc*-cluster were sequentially deleted in the marker free NID598 background. For this, the required targeting cassettes were constructed via directional USER cloning of the respective approximately 1500 bp long targeting fragments into the pU2002-A vector, as described by Hansen *et al*.^18^. The used primers are listed in Supplementary File, Table 2 and each includes a 2-deoxyuridin to allow for the formation of 3′-overhangs upon treatment with Uracil-Specific Excision Reagent (USER). Gel-purified PCR amplicons were USER-cloned into PacI/Nt.BbvCI digested pU2002-A vector. The inserts in the resulting plasmids were Sanger sequenced (GATC, Germany) and the targeting cassette, including the *AfpyrG* marker, was liberated by *SwaI* digesting. The digested cassettes were sequentially transformed into the marker-free NID598 strain to create the NID2252 strain which lacks the asperthecin, monodictyphenone/emodin and sterigmatocystin gene clusters (see Supplementary File, Table 1). The introduced deletions were validated by diagnostic PCR reactions bridging the deleted area of the genome.

### Synthetic genes

The coding sequences for *OKS* (Genbank accession no.: Q3L7F5) from *Aloe arborescens* was *de novo* synthesized by GenScript (Hong Kong, PRC) both with the original codon usage and as codon optimized for expression in *Aspergillus*. Codon optimization was performed via GenScript’s OptimumGene^Tm^ algorithm. *ZhuI* (Genbank accession no.: Q9F6D3) and *ZhuJ* (Genbank accession no.: Q9F6D2) from *Streptomyces* sp. R1128 were codon optimized for expression in *Aspergillus* and *de novo* synthesized by GenScript (Hong Kong, PRC). The *UGT2* (Genbank accession no.:KY860725) from *D*. *coccus* was codon optimized for expression in *Aspergillus* and *de novo* synthesized by GenScript.

The delivered synthetic genes were amplified by PCR using the X7 DNA polymerase and the primers listed in Supplementary File, Table 2. The amplified coding sequences were gel-purified by using the GFX PCR DNA and Gel Band Purification Kits (Sigma-Aldrich, Copenhagen, Denmark) and USER cloned into vectors targeting predetermined loci (IS = integration site) in the *A*. *nidulans* genome. *OKS* was cloned into the pIS5 vector, *ZhuI* into the pIS6 vector, *ZhuJ* into the pIS7 vector and *UGT2* into the pIS4 vector^18^. The four vectors all feature a PacI/Nt.BbvCI USER cloning site situated between the *PgpdA* promoter and *TtrpC* terminator, both elements originating from *A*. *nidulans*. Following validation of the vector constructions by sequencing of the inserts, the individual expression cassettes, flanked by up- and downstream targeting sequences and the *AfpyrG* marker, were liberated by SwaI digestion. The resulting fragments were gel-purified using the GFX kit. The expression cassettes were sequentially introduced into the NID2252 strain by protoplast transformation as described by Nielsen *et al*.^32^, resulting in the strains listed in Supplementary File, Table 1. Following transformation, PCR-based verification of targeted integration and targeted Sanger sequencing of the respective expression cassettes, the *AfpyrG* marker was eliminated by 5-FOA counter selection to allow for further genetic engineering of the strain.

### Chemical analysis

For chemical analysis, the strains were grown in triplicates on solid MM + Ura + Uri + Arg medium for 7 days at 37 °C in darkness. Approximately 7 mL of MM agar with fungal biomass was extracted two times with 30 mL 69% (v/v) ethyl acetate, 30% (v/v) *n*-butanol and 1% (v/v) formic acid for 1 hour at room temperature. The organic phase was decanted to new tubes and the solvent was evaporated to dryness under a flow of nitrogen gas. The pellet was resuspended in 400 μL of MeOH + 1% formic acid aided by ultrasonication for 20 minutes. The sample was centrifuged at 15,000 x *g* for 5 minutes and 300 μL were transferred to HPLC vials. HPLC-HRMS/MS analyses were performed on an Agilent 1200 HPLC coupled to a Bruker micrOTOF-Q II mass spectrometer equipped with an electrospray ionization source. Mass spectra were acquired in positive- and negative-ion mode at 200 °C drying temperature, a capillary voltage of 4100 V or 4000 V, respectively, a nebulizer pressure of 2.0 bar and a drying gas flow of 7 L/min. A solution of sodium formate clusters was automatically injected at the beginning of each run to enable internal mass calibration. Chromatographic separation was obtained on a Luna C_18_(2) column (150 × 4.6 mm, 3 μm, 100 Å, Phenomenex, Værløse, Denmark) maintained at 40 °C. The aqueous eluent (A) consisted of water/acetonitrile (95:5, v/v) and the organic eluent (B) consisted of water/acetonitrile (5:95, v/v); both acidified with 0.1% formic acid. The following linear gradient elution profile was used: 0 min, 0% B; 20 min, 100% B; 29 min 100% B; 30 min, 0% B. The flow rate was maintained at 0.8 mL/min and 7 min equilibration was used. For comparison of production levels across samples the MS base peak ion counts were normalized to linoleic acid levels, a measure of extracted biomass, in the individual samples. Identification of flavokermesic acid, kermesic acid, dcII, carminic acid, and linoleic acid was based on comparison of retention time and MS/MS fragmentation patterns of authentic reference samples isolated from *D*. *coccus*^7^ or purchased from Sigma-Aldrich (linoleic acid). SEK4 and SEK4b was identified by HPLC-HRMS/MS fragmentation patterns^19^, while the identity of mutactin was based on comparison of HRMS and UV spectra with previously published data^34^.

### Structural elucidation of dehydro-SEK4, dehydro-SEK4b

Dehydro-SEK4 and dehydro-SEK4b were identified using a hyphenated HPLC-HRMS-SPE-NMR system^35^, which consisted of the above HPLC-HRMS equipment, connected to a Spark Holland Prospekt-2 solid-phase extraction unit, a Knauer Smartline K-120 pump for post-column dilution with water, and a Gilson 215 liquid handler for automated filling of 1.7-mm NMR tubes. NMR spectra were recorded at 300 K with a Bruker Avance III 600 MHz spectrometer (^1^H operating frequency 600.13 MHz) equipped with a cryogenically cooled 1.7-mm TCI probe using methanol-*d*_4_ or acetonitrile-*d*_3_ (99.8 atom % of deuterium) as solvent. Chromatographic separation was obtained using the same column and solvents as described above with the following gradient elution profile: 0 min, 5% B; 40 min, 60% B; 42 min 100% B; 49 min, 100% B; 50 min 5% B. The flow rate was maintained at 0.5 mL/min with a post-column dilution of water at a flow rate of 1 mL/min. Structure elucidation was based on 1-D (^1^H) and 2-D (HSQC, HMBC, DQF-COSY) spectra, and assignments were in accordance with published data^20^, with a notable deuterium exchange observed at position C-2 in methanol-*d*_4_. As expected, this exchange was not observed when eluting the metabolites in acetonitrile-*d*_3_.

## Electronic supplementary material

Supplementary information
