# Supplementary material for: Heterologous production of the widely used natural food colorant carminic acid in Aspergillus nidulans
Source: Sci Rep. 2018 Aug 27;8:12853. doi: 10.1038/s41598-018-30816-9 (PMC6110711; doi:10.1038/s41598-018-30816-9)
Supplement: Supplementary file 1 — Supplementary information [file 41598_2018_30816_MOESM1_ESM.docx]

**Supplementary information**

# *Heterologous production of the widely used natural food colorant carminic acid in Aspergillus nidulans*

Rasmus J.N. Frandsen^1^*, Paiman Khorsand-Jamal^1,2,3^, Kenneth T. Kongstad^4.^**, Majse Nafisi^2,5^, Rubini M. Kannangara^2,5^, Dan Staerk^4^, Finn Okkels^2,6^, Kim Binderup^2,7^, Bjørn Madsen^2^, Birger Lindberg Møller^5,8^, Ulf Thrane^1,9^ & Uffe H. Mortensen^1^

^1^Section for Synthetic Biology, Department of Biotechnology and Biomedicine, The Technical University of Denmark, Kongens Lyngby, Denmark. ^2^Chr. Hansen A/S, Hoersholm, Denmark. ^3^Novo Nordisk A/S, Maaloev, Denmark. ^4^Department of Drug Design and Pharmacology, University of Copenhagen, Copenhagen, Denmark. ^5^Plant Biochemistry Laboratory, Department of Plant and Environmental Sciences, University of Copenhagen, Frederiksberg, Denmark. ^6^Present address: Actabio ApS, Roskilde, Denmark. ^7^Present address: DSM Nutritional Products, Kaiseraugst, Switzerland. ^8^Center for Synthetic Biology, University of Copenhagen, Frederiksberg, Denmark. ^9^Present address: Department of Energy Performance, Indoor Environment and Sustainability, Danish Building Research Institute, Aalborg University Copenhagen, Copenhagen, Denmark. *E-mail: [rasf@bio.dtu.dk](mailto:rasf@bio.dtu.dk), corresponding author synthetic biology. **E-mail: [kenneth.kongstad@sund.ku.dk](mailto:kenneth.kongstad@sund.ku.dk), corresponding author analytical chemistry

**Supplementary Table 1:** The *Aspergillus nidulans* strains used in the current study.

| Strain | Genotype | Origin |
| --- | --- | --- |
| *NID1* | *nkuA*Δ *argB2*, *pyrG89*, *veA1* | Nielsen *et al.*, 2008^36^. |
| *NID598* | NID1 with *wA*∆ and *yA*∆ | This study |
| NID2252 (Δcluster) | NID598 with *wA*Δ (AN8209), *yA*Δ (AN6635), *apt*Δ (AN6000-AN6002), *mdp*Δ (AN10023-AN10021), *stc*Δ (AN7804-AN7825) | This study |
| *OKS_Nativ* | NID2252 with IS5:*OKS_orig* | This study |
| *OKS_Opt* | NID2252 with IS5:*OKS_Opt* | This study |
| *ZhuI* | NID2252 with IS6:*ZhuI* | This study |
| *ZhuJ* | NID2252 with IS7:*ZhuJ* | This study |
| *ZhuI+ZhuJ* | NID2252 with IS6:*ZhuI*, IS7:*ZhuJ* | This study |
| *OKS+ZhuI* | NID2252 with IS5:*OKS_orig,* IS6:*ZhuI* | This study |
| *OKS+ZhuJ* | NID2252 with IS5:*OKS_orig*, IS7:*ZhuJ* | This study |
| *OKS+ZhuI+ZhuJ* | NID2252 with IS5:*OKS_orig,* IS6:*ZhuI*, IS7:*ZhuJ* | This study |
| *UGT2* | NID2252 with IS4:*UGT2* | This study |
| *OKS+ZhuI+ZhuJ+UGT2* | NID2252 with IS5:*OKS_orig,* IS6:*ZhuI*, IS7:*ZhuJ*, IS4:*UGT2* | This study |

**Supplementary Table 2:** Primers used for construction of the presented carminic acid *Aspergillus nidulans* cell factory.

| Primer name | Sequence (5’ to 3’) | Purpose |
| --- | --- | --- |
| M1 | **catggcaattcccggggatc**GCCGGCAATTCTTTTTAGGTAGC | Upstream fragment of *AfpyrG* bipartial marker for deletion |
| M2 | CCAGAAGCAGTACACGGC |  |
| M3 | GTTGTCTGCTTGCGCTTCTTC | Downstream fragment of *AfpyrG* bipartial marker for deletion |
| M4 | **catggtggtcagctggaat**TCCTCCGCCATTTCTTATTCCC |  |
| AN*wA*-dl-Up-F | GGAAGAAGGTCGCATACCA | Deletion of *wA* PKS (AN8209) via bipartial marker system |
| AN*wA*-dl-Up-Rad | **gatccccgggaattgccatg**GATCAGGAGAAGGAGAGTCAAG |  |
| AN*wA*-dl-Dw-Fad | **aattccagctgaccaccatg**GGCGAAAAGGCAAAGGAGC |  |
| AN*wA*-dl-Dw-R | GCTAGAAAAGGCAAGGGAGG |  |
| Del-*yA*-5’-F | GTGGGTTGAACCGCTTACTCAG | Deletion of *yA* laccase (AN6635) via bipartial marker system |
| Del-*yA*-5’-R | **gatccccgggaattgccatg**CCCGGAGGAATCAAAATGACGC |  |
| Del-*yA*-3’F | **aattccagctgaccaccatg**GTTTGGGATTCTTAGGTGAGCTC |  |
| Del-*yA*-3’-R | CCTCCCTGGCGTATACACAAAC |  |
| AN_APTcluster-Dl-Up-FU | GGGTTTAAUGAGGAGCAGAGGATGCGG | Deletion of *Apt*-cluster (AN6000 to AN6002) via USER system |
| AN_APTcluster-Dl-Up-RU | GGACTTAAUGTAGTGGTGGTGCTGGTG |  |
| AN_APTcluster-Dl-Dw-FU | GGCATTAAUCGCGTGGAATTTGGAAGAGAG |  |
| AN_APTcluster-Dl-Dw-RU | GGTCTTAAUGTGCTCGGGGACGTGAAAG |  |
| AN_MDPcluster-Dl-Up-FU | GGGTTTAAUGGTCGTCTGTCAAGGAGTTG | Deletion of *mdp*-cluster (AN10023 to AN10021) via USER system |
| AN_MDPcluster-Dl-Up-RU | GGACTTAAUGCAGTGCTGTATATGGGTCTTG |  |
| AN_MDPcluster-Dl-Dw-FU | GGCATTAAUGAGTTTGTGAGATGTTCAGGATGG |  |
| AN_MDPcluster-Dl-Dw-RU | GGTCTTAAUGAGGTGAAGGACACAGCG |  |
| AN_STCcluster-Dl-Up-FU | GGGTTTAAUCGCAGAGACTAGGACACAAGTG | Deletion of via *stc*-cluster (AN7804 to AN7825) via USER system |
| AN_STCcluster-Dl-Up-RU | GGACTTAAUGCGGCGATCTGTGGTAGAG |  |
| AN_STCcluster-Dl-Dw-FU | GGCATTAAUGCCAGCATATTCAAACCCAGTC |  |
| AN_STCcluster-Dl-Dw-RU | GGTCTTAAUCACACAACCAACCTCCGATC |  |
| OKS_Orig-F | AGAGCGAUATGAGTTCACTCTCCAACGCTTCC | Expression of *OKS* in *A. nidulans* |
| OKS_Orig-R | TCTGCGAUTCACATGAGAGGCAGGCTGTG |  |
| OKS_Opt-F | AGAGCGAUATGAGTAGTTTATCAAATGCCAGTC | Expression of *OKS* in *A. nidulans* |
| OKS_Opt-R | TCTGCGAUTCACATCAATGGCAAGGAA |  |
| ZhuI-F | AGAGCGAUGAGACACGTTGAACACA | Expression of *ZhuI* in *A. nidulans* |
| ZhuI-R | TCTGCGAUTTATGCAGTTACGGTACCA |  |
| ZhuJ-F | AGAGCGAUGTCCGGTAGAAAGACCTT | Expression of *ZhuJ* in *A. nidulans* |
| ZhuJ-R | TCTGCGAUTTAATCTTCTTCTTCTTGTTCG |  |
| UGT2_Opt-F | AGAGCGAUATGGAGTTTCGCTTGCTTATCCT | Expression of *UGT2* in *A. nidulans* |
| UGT2_Opt-R | TCTGCGAUTTAATTCTTCTTCAACTTTTCCGACTTAG |  |

**Supplementary Figure 1:** Phenotype of the *Aspergillus nidulans* reference strain (NID1) and the constructed ‘cluster deletion’ *A. nidulans* strain NID2252 after 7 days of growth on minimal medium at 37 ^o^C in darkness.


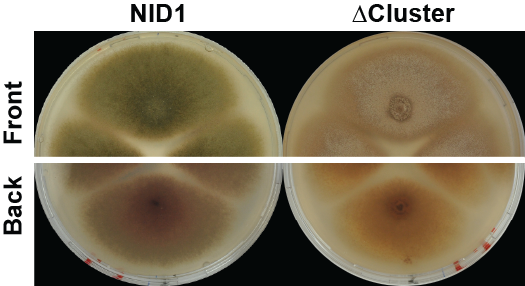


**Supplementary Figure 2: HRMS/MS analysis of SEK4 and SEK4b**

HPLC-ESI(-)-HRMS/MS spectra of SEK4 (A) and SEK4B (B). For both compounds, the [2M-H]^-^  ion was isolated for fragmentation, yielding product ions, corresponding to previously reported values^1^.


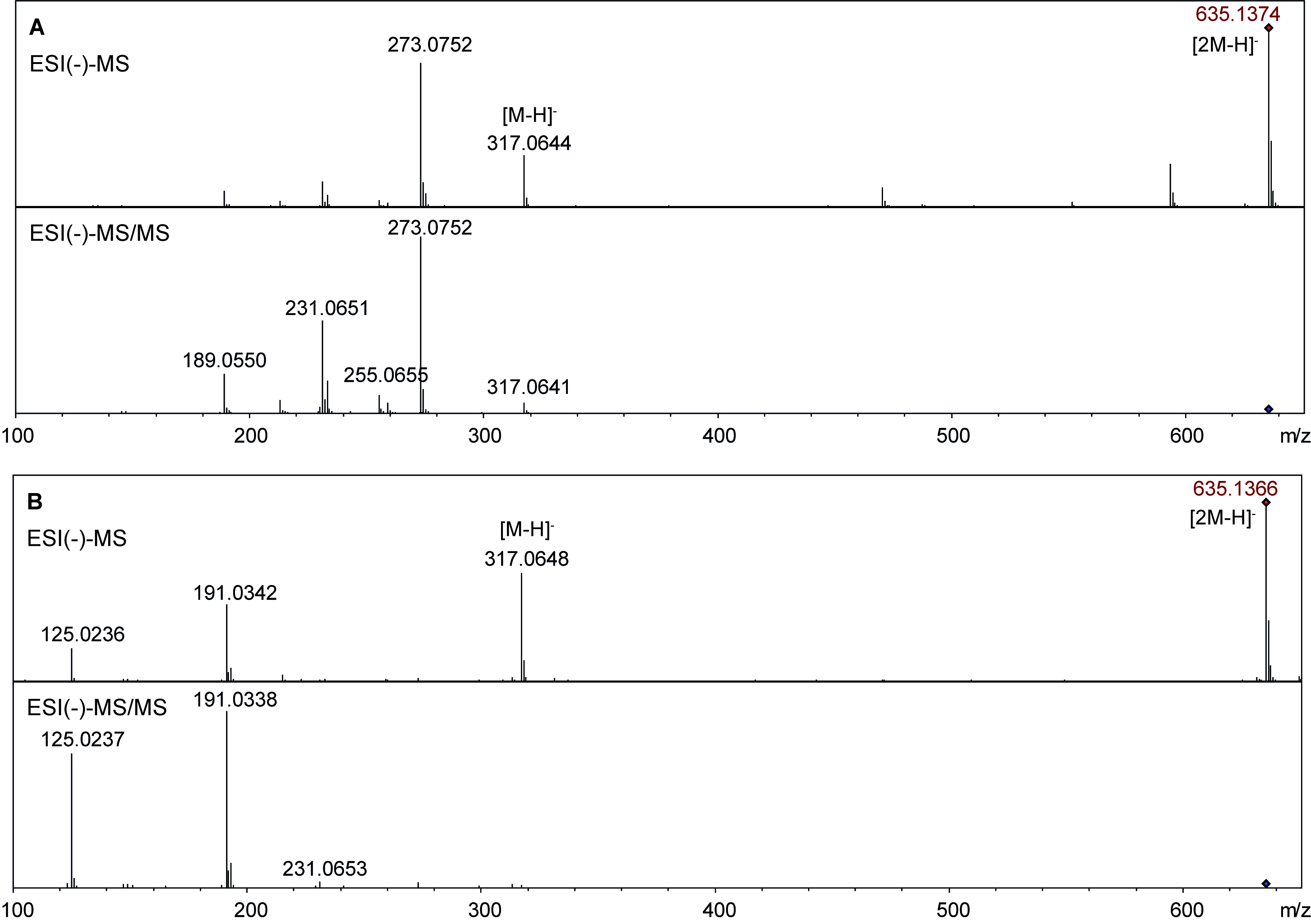


**Supplementary Figure 3: HRMS/MS analysis of flavokermesic acid**

HPLC-ESI(-)-HRMS/MS spectra of Flavokermesic acid (FK). The [M-H]^-^  ion was isolated for fragmentation, yielding product ions, corresponding to previously reported values^2^.


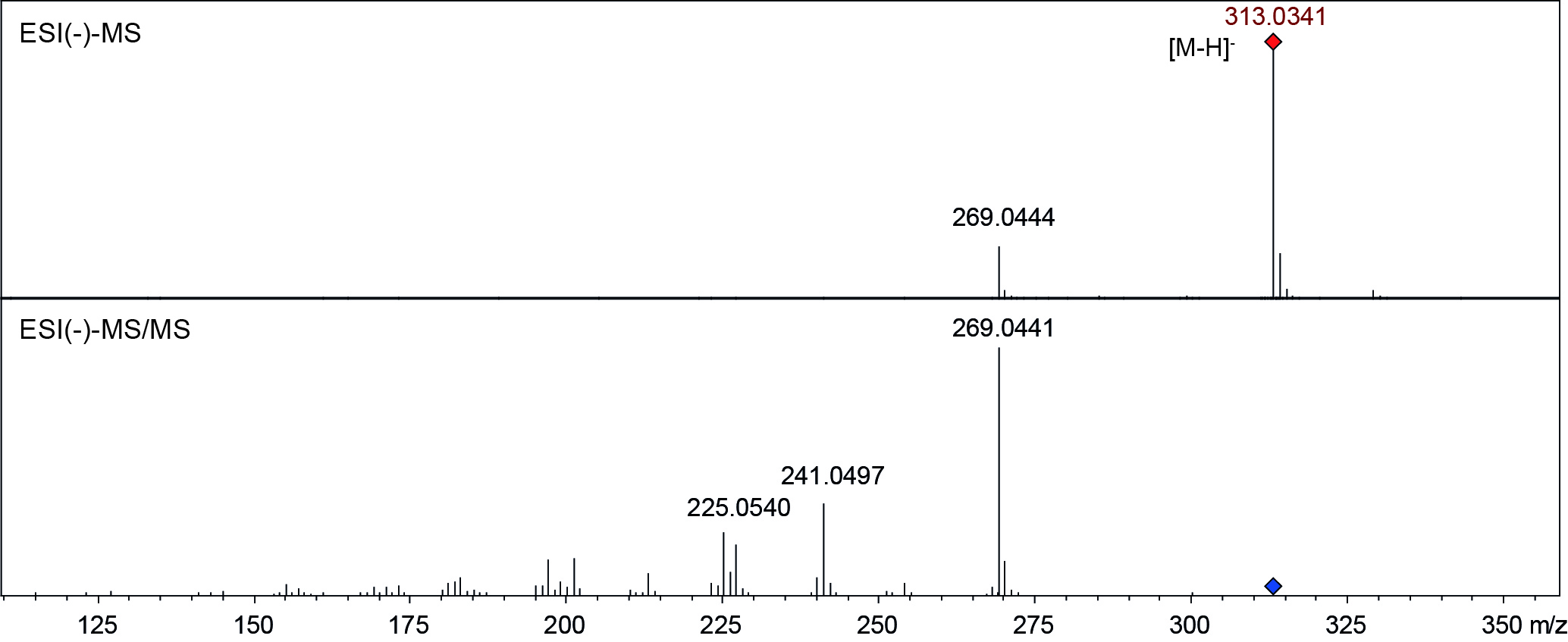


**Supplementary Figure 4: HRMS/MS and UV-VIS analysis of mutactin**

HPLC-ESI(+)-HRMS/MS spectra of mutactin (A) and UV spectrum acquired in during HPLC-HRMS analysis (B). HPLC-HRMS (303.0867; [C_16_H_15_O_6_]^+^ ΔM=-1.2 ppm) and UV spectrum corresponds to previously reported values^3,4^.

**
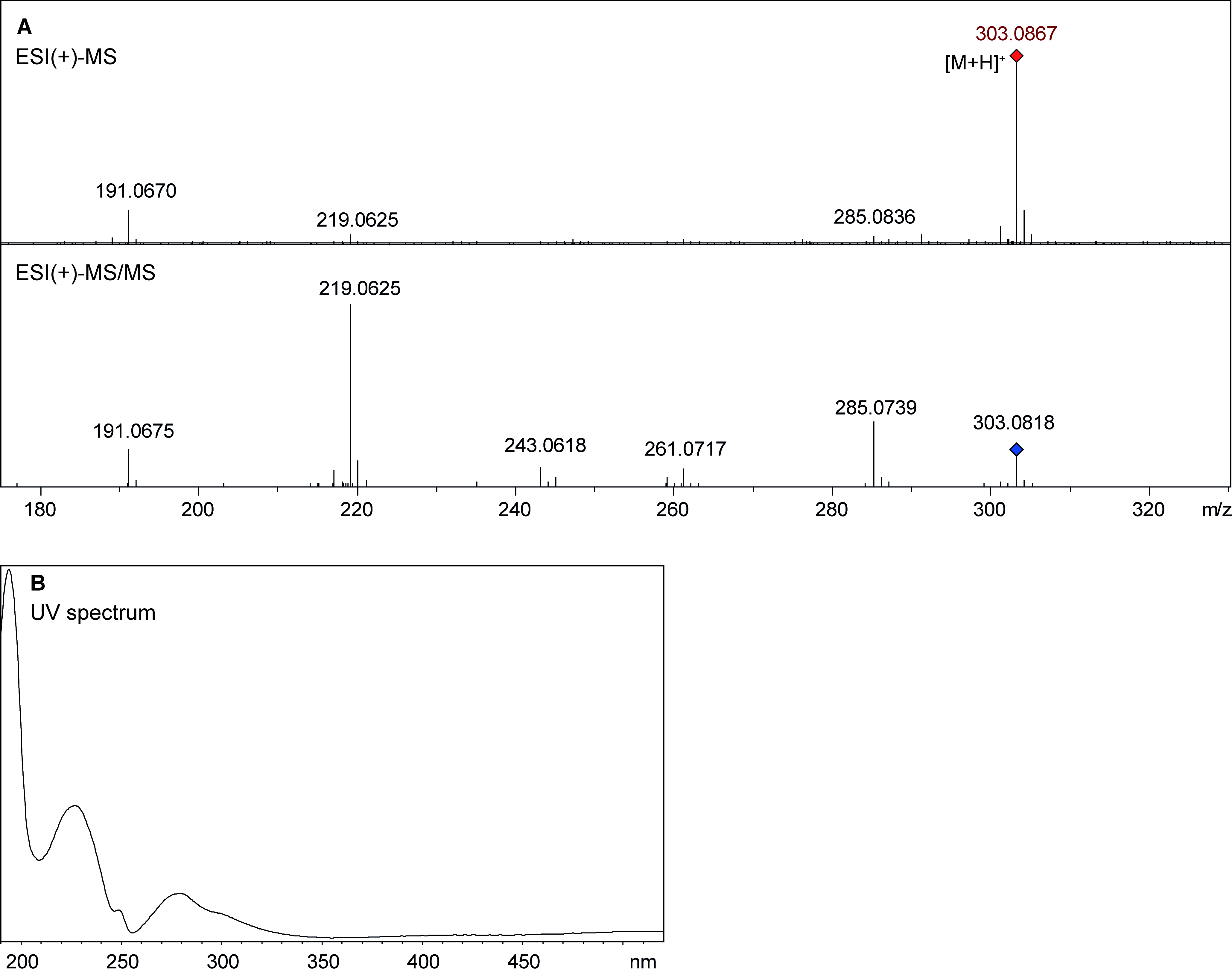
**

**Supplementary Figure 5: HRMS/MS analysis of dehydro-SEK4 and dehydro-SEK4b**

HPLC-ESI(-)-HRMS/MS spectra of dehydroSEK4 (A) and dehydroSEK4B (B). For dehydro-SEK4 the [M-H]^-^  ion was isolated for fragmentation, while for dehydro-SEK4B, the more abundant [2M-H]^-^ ion was isolated^5^.


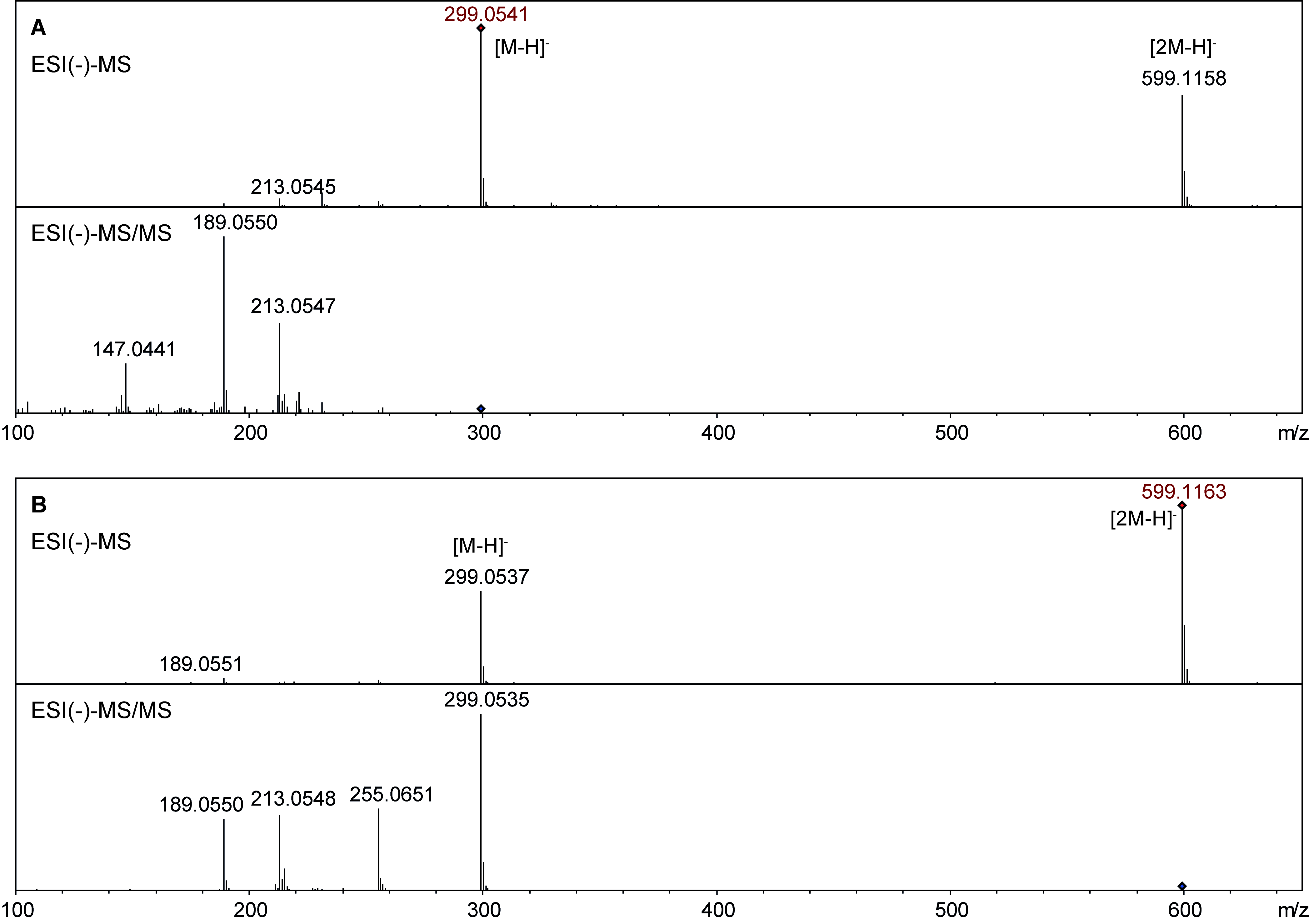


**Supplementary Figure 6: ^1^H NMR spectrum of dehydro-SEK4**

^1^H NMR spectrum of dehydro-SEK4 acquired in HPLC- HRMS-SPE-NMR mode (600 MHz, methanol-*d*_4_, 300 K) with notably deuterium exchange of H-2.


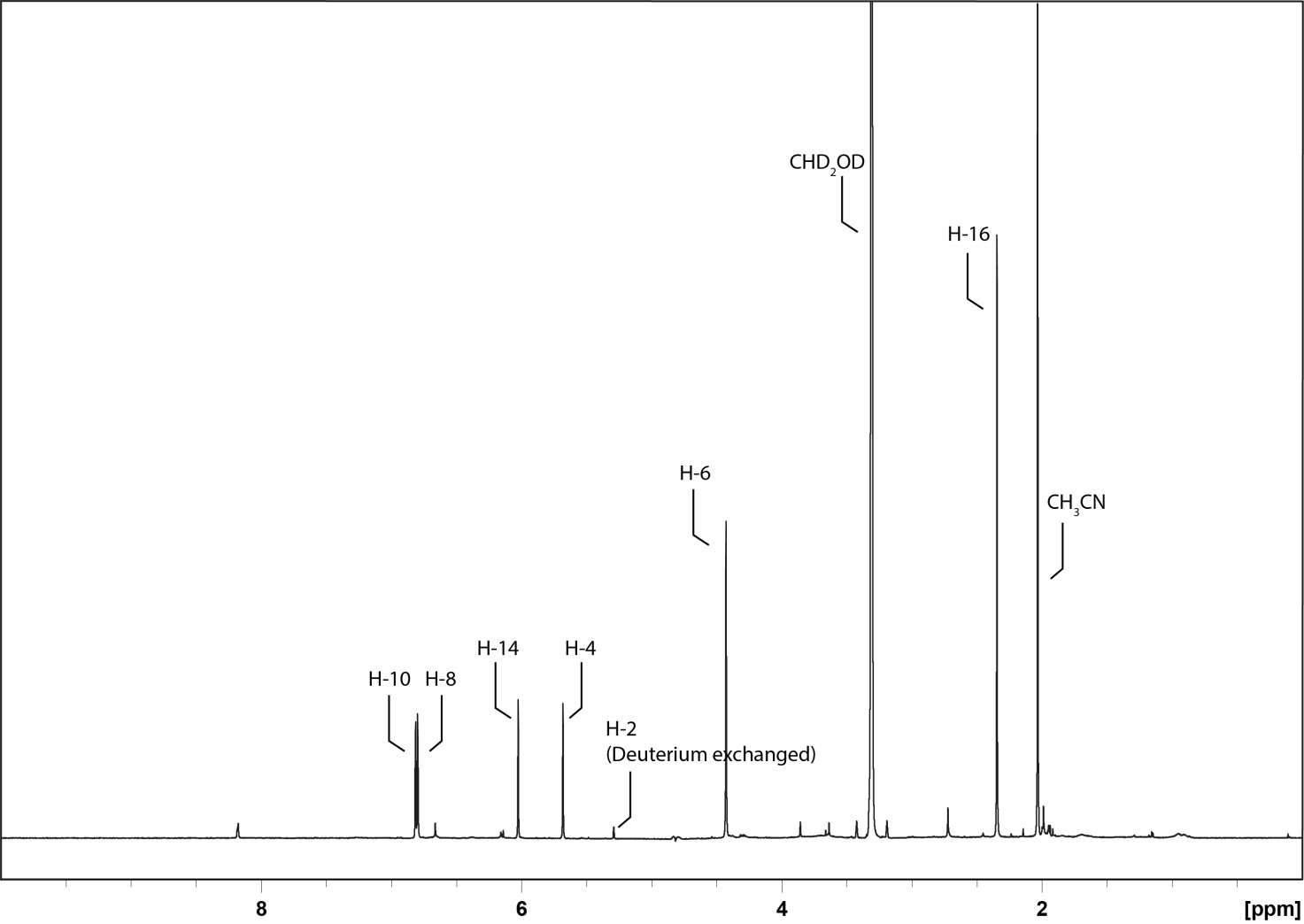


**Supplementary Figure 7: HSQC spectrum of dehydro-SEK4**

HSQC spectrum of dehydro-SEK4 acquired in HPLC- HRMS-SPE-NMR mode (600 MHz, methanol-*d*_4_, 300 K).


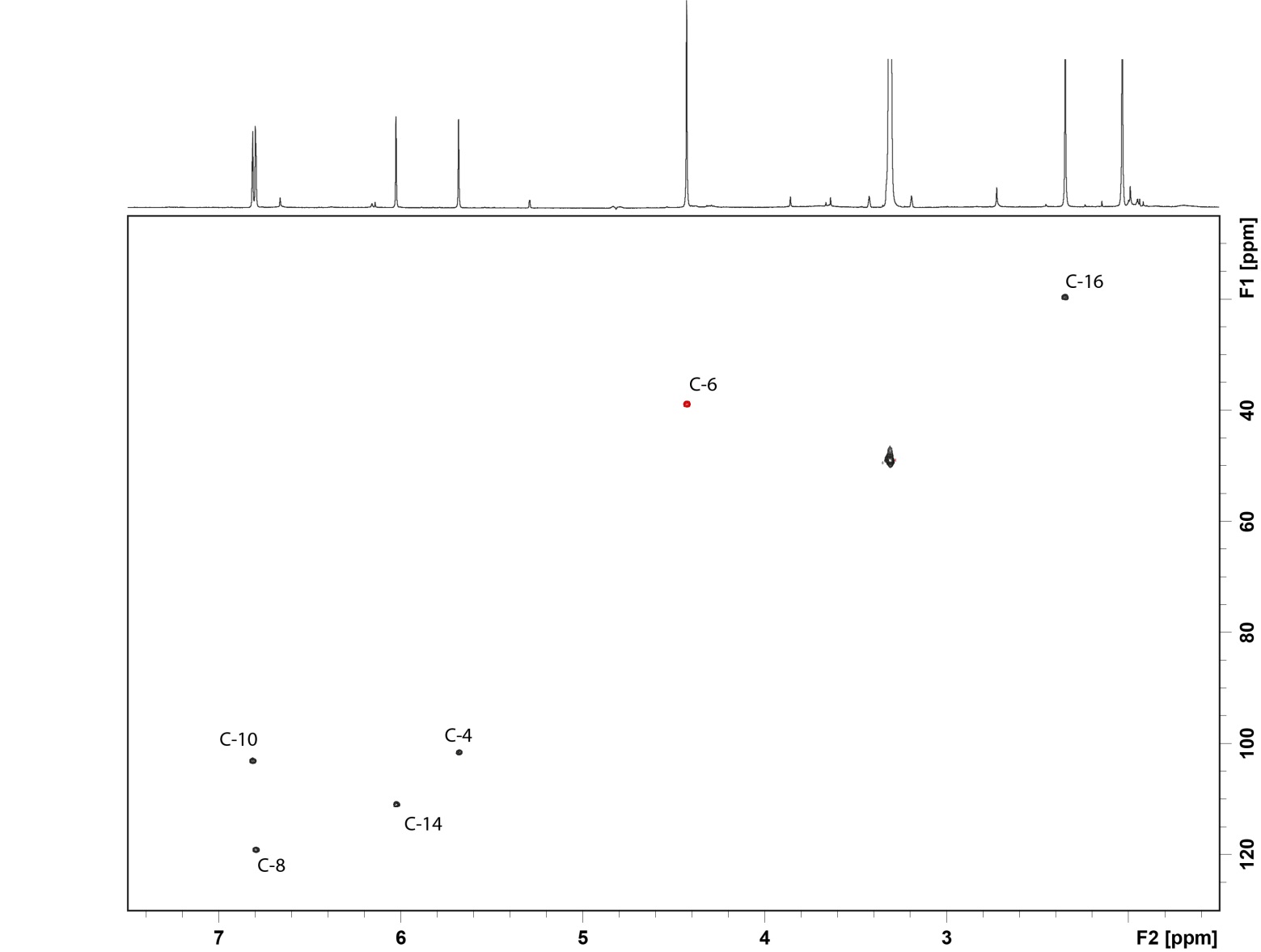


**Supplementary Figure 8: HMBC spectrum of dehydro-SEK4**

HSQC spectrum of dehydro-SEK4 acquired in HPLC- HRMS-SPE-NMR mode (600 MHz, methanol-*d*_4_, 300 K).


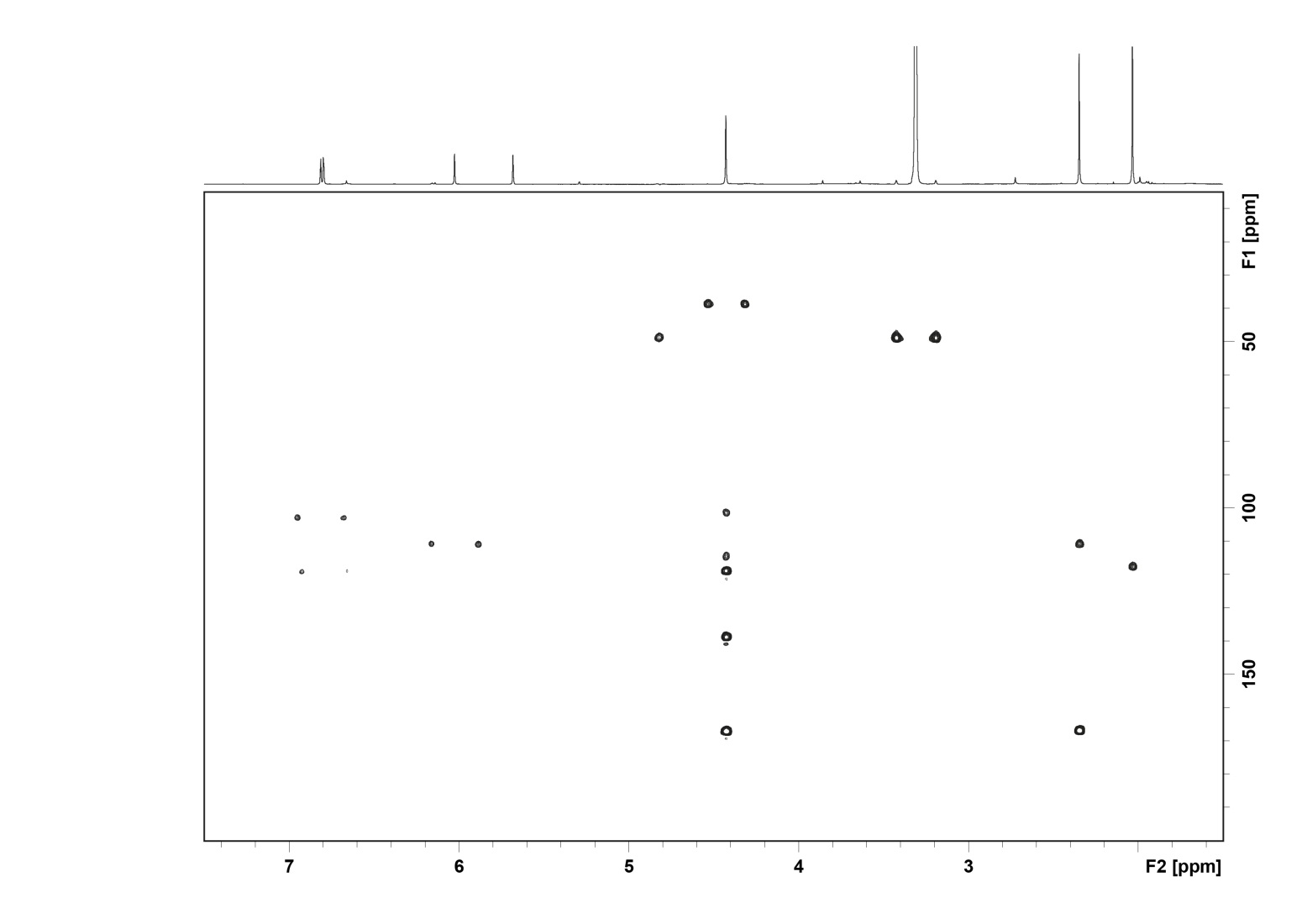


**Supplementary Figure 9: ^1^H NMR spectrum of dehydro-SEK4b**

^1^H NMR spectrum of dehydro-SEK4B acquired in HPLC- HRMS-SPE-NMR mode (600 MHz, methanol-*d*_4_, 300 K) with notably deuterium exchange of H-2.


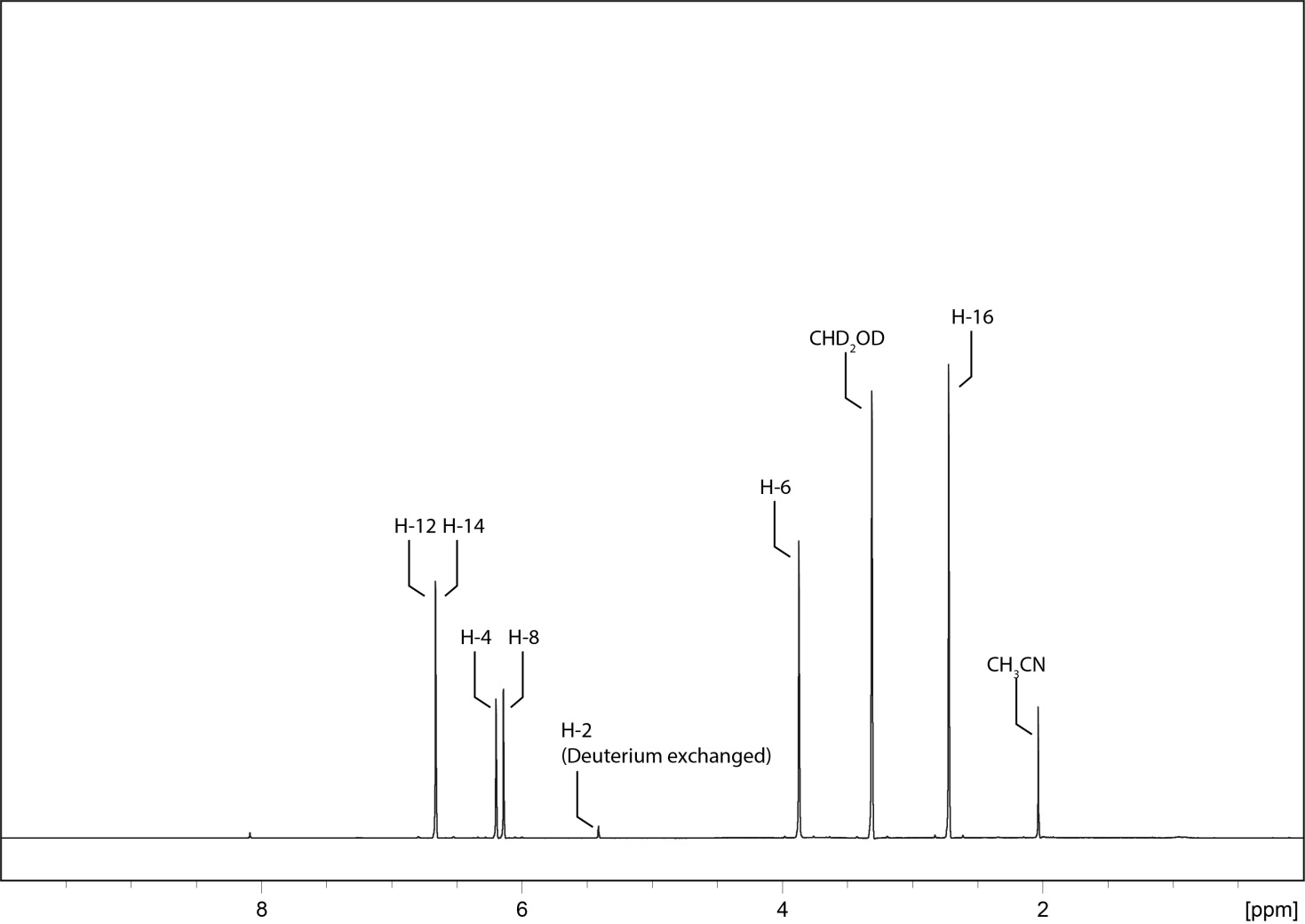


**Supplementary Figure 10: HSQC spectrum of dehydro-SEK4b**

HSQC spectrum of dehydro-SEK4B acquired in HPLC- HRMS-SPE-NMR mode (600 MHz, methanol-*d*_4_, 300 K).


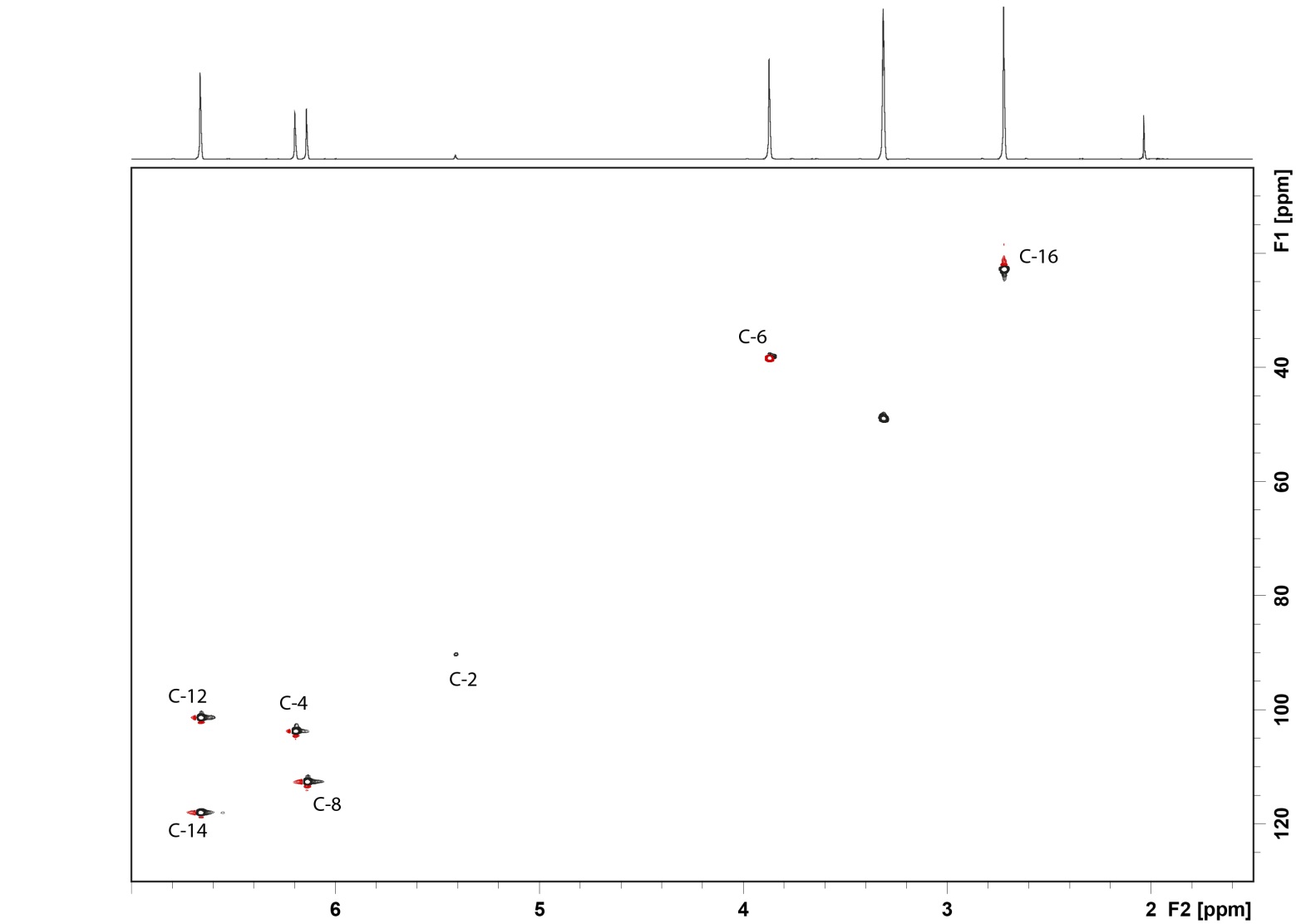


**Supplementary Figure 11: HMBC spectrum of dehydro-SEK4b**

HSQC spectrum of dehydro-SEK4B acquired in HPLC- HRMS-SPE-NMR mode (600 MHz, methanol-*d*_4_, 300 K).


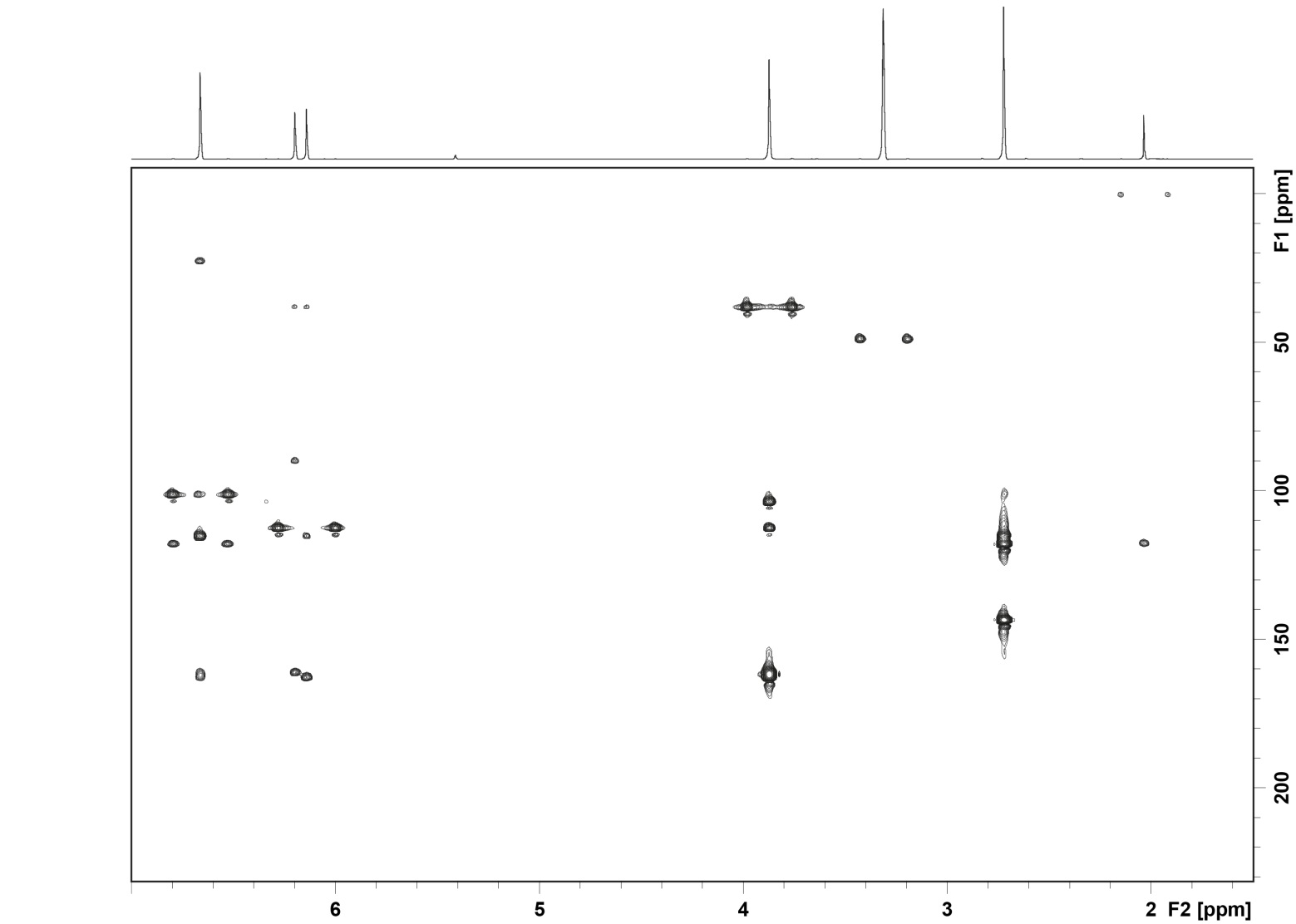


**Supplementary Figure 12: The observed folding modes.**

The intramolecular reactions leading to the formation of SEK4, SEK4b, dehydro-SEK4, dehydro-SEK4b, mutactin and flavokermesic acid anthrone.

**
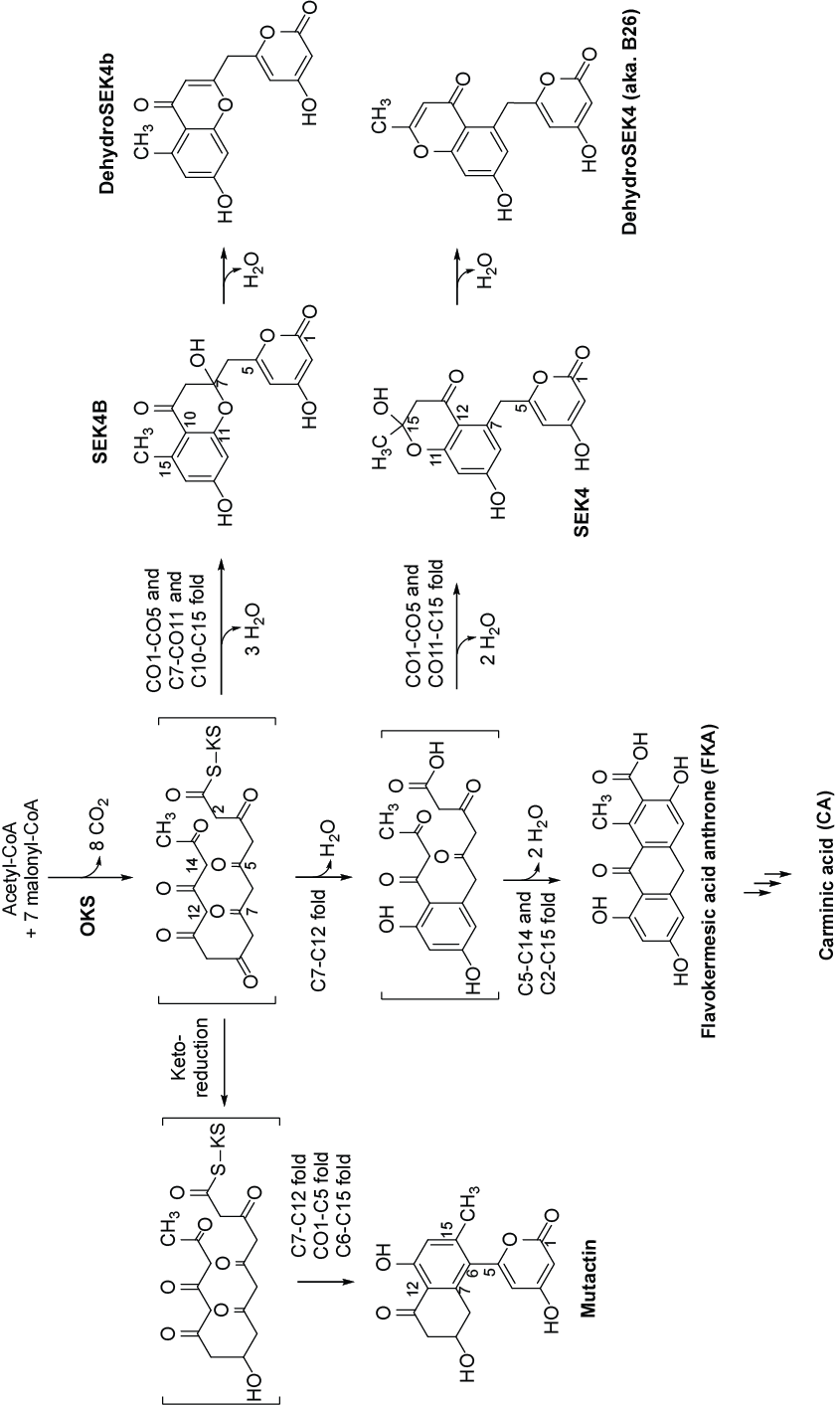
**

**Supplementary Figure 13: HRMS/MS analysis of carminic acid**

HPLC-ESI(-)-HRMS/MS spectra of Carminic acid (CA). The [M-H]^-^  ion was isolated for fragmentation, yielding product ions, corresponding to previously reported values^2,6,7^.


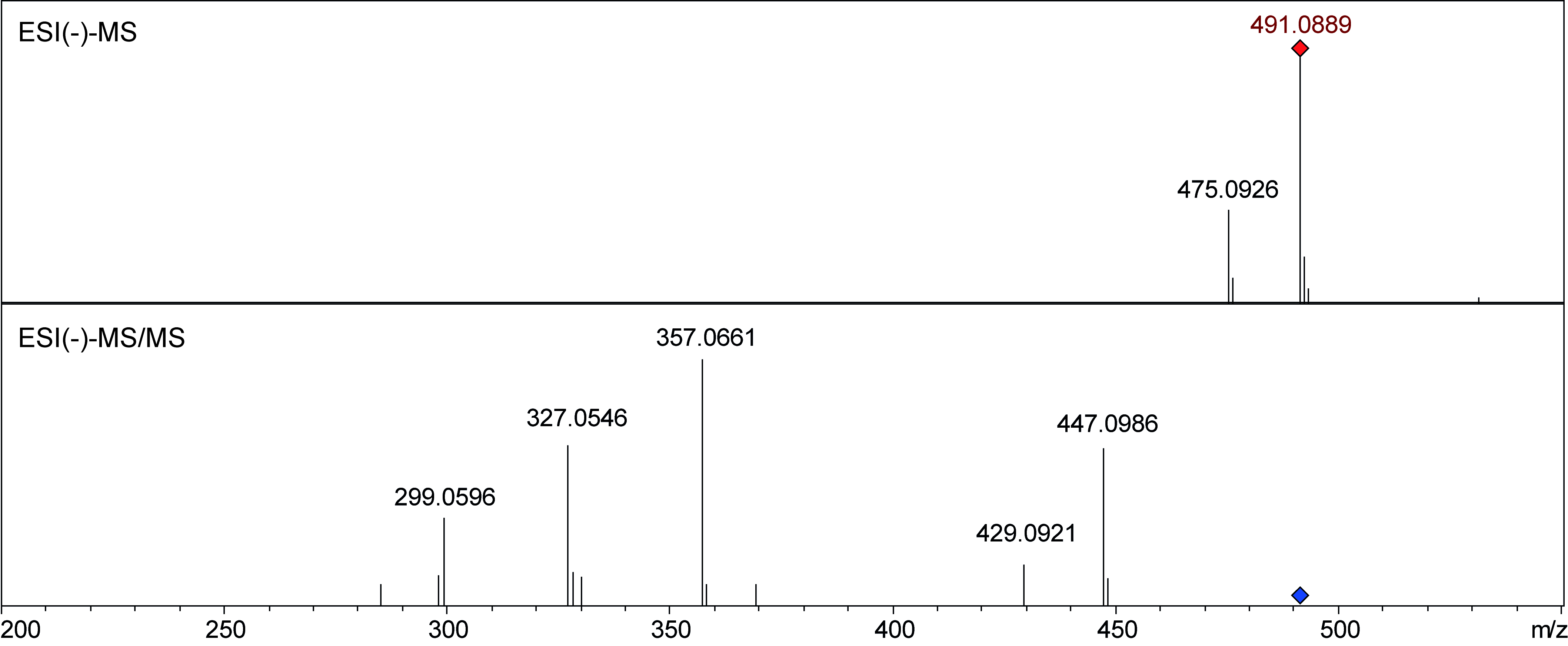


**Supplementary Figure 14: HRMS/MS characteristics of dcII and dcII analogue.**

HPLC-ESI(-)-HRMS/MS analysis of dcII and dcII analogue reveals similar cross-ring cleavage fragmentation patterns, characteristic of C-glucosides, with notable differences in intensities corresponding to those reported by Jarosz and co-workers^8^ for dc1 and dcII.


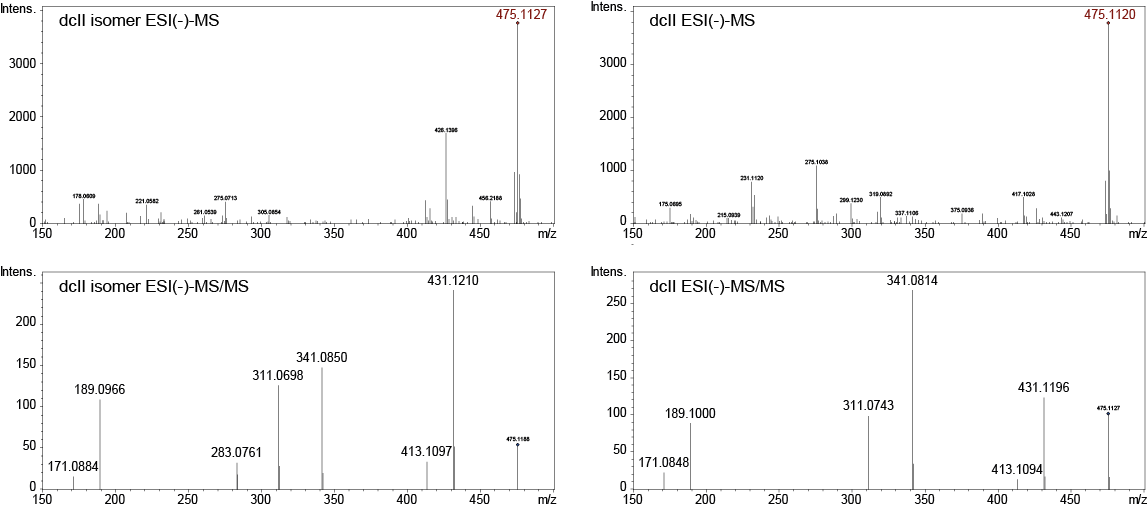


**References for Supplementary information:**

1. Karppinen, K., Hokkanen, J., Mattila, S., Neubauer, P. & Hohtola, A. Octaketide-producing type III polyketide synthase from *Hypericum perforatum* is expressed in dark glands accumulating hypericins. *FEBS J.* **275,** 4329–42 (2008).

2. Rasmussen, S. A. *et al.* On the biosynthetic origin of carminic acid. *Insect Biochem. Mol. Biol.* **96,** 51–61 (2018).

3. Javidpour, P., Korman, T. P., Shakya, G. & Tsai, S.-C. Structural and biochemical analyses of regio- and stereospecificities observed in a type II polyketide ketoreductase. *Biochemistry* **50,** 4638–49 (2011).

4. Das, A. & Khosla, C. Biosynthesis of Aromatic Polyketides in Bacteria. *Acc. Chem. Res.* **42,** 631–639 (2009).

5. Xiang, L., Kalaitzis, J. A. & Moore, B. S. EncM, a versatile enterocin biosynthetic enzyme involved in Favorskii oxidative rearrangement, aldol condensation, and heterocycle-forming reactions. *Proc. Natl. Acad. Sci.* **101,** 15609–15614 (2004).

6. Schmitt, P., Günther, H., Hägele, G. & Stilke, R. A ^1^H and ^13^ C NMR Study of Carminic Acid. *Org. Magn. Reson.* **22,** 446–449 (1984).

7. Stathopoulou, K., Valianou, L., Skaltsounis, A.-L., Karapanagiotis, I. & Magiatis, P. Structure elucidation and chromatographic identification of anthraquinone components of cochineal (*Dactylopius coccus*) detected in historical objects. *Anal. Chim. Acta* **804,** 264–72 (2013).

8. Lech, K., Witkoś, K., Wileńska, B. & Jarosz, M. Identification of unknown colorants in pre-Columbian textiles dyed with American cochineal (*Dactylopius coccus* Costa) using high-performance liquid chromatography and tandem mass spectrometry. *Anal. Bioanal. Chem.* **407,** 855–867 (2015).
